# Developmentally regulated *HEART STOPPER*, a mitochondrially targeted L18 ribosomal protein gene, is required for cell division, differentiation, and seed development in *Arabidopsis*

**DOI:** 10.1093/jxb/erv296

**Published:** 2015-06-23

**Authors:** Hongyu Zhang, Ming Luo, Robert C. Day, Mark J. Talbot, Aneta Ivanova, Anthony R. Ashton, Abed M. Chaudhury, Richard C. Macknight, Maria Hrmova, Anna M. Koltunow

**Affiliations:** ^1^Rice Research Institute of Sichuan Agricultural University, Chengdu, Sichuan 611130, PR China; ^2^CSIRO Agriculture Flagship, PO Box 1600, ACT 2601, Australia; ^3^Department of Biochemistry, University of Otago, Dunedin, New Zealand; ^4^ Present address: ARC Centre of Excellence in Plant Energy Biology, The University of Western Australia, WA 6009, Australia; ^5^ Present address: VitaGrain, 232 Orchard Road, Level 9, Suite 232, Faber House, 238854Singapore; ^6^Australian Centre for Plant Functional Genomics, School of Agriculture, Food and Wine, University of Adelaide, Glen Osmond, SA 5064, Australia; ^7^CSIRO Agriculture Flagship, PO Box 350, Glen Osmond, SA 5064, Australia

**Keywords:** Cellularization, development, differentiation, embryo, endosperm, lethality, mitochondria, ribosome, ros, seed.

## Abstract

Evidence is presented for the role of a mitochondrial ribosomal (mitoribosomal) L18 protein in cell division, differentiation, and seed development after the characterization of a recessive mutant, *heart stopper* (*hes*). The *hes* mutant produced uncellularized endosperm and embryos arrested at the late globular stage. The mutant embryos differentiated partially on rescue medium with some forming callus. *HES* (At1g08845) encodes a mitochondrially targeted member of a highly diverged L18 ribosomal protein family. The substitution of a conserved amino residue in the *hes* mutant potentially perturbs mitoribosomal function via altered binding of 5S rRNA and/or influences the stability of the 50S ribosomal subunit, affecting mRNA binding and translation. Consistent with this, marker genes for mitochondrial dysfunction were up-regulated in the mutant. The slow growth of the endosperm and embryo indicates a defect in cell cycle progression, which is evidenced by the down-regulation of cell cycle genes. The down-regulation of other genes such as *EMBRYO DEFECTIVE* genes links the mitochondria to the regulation of many aspects of seed development. *HES* expression is developmentally regulated, being preferentially expressed in tissues with active cell division and differentiation, including developing embryos and the root tips. The divergence of the L18 family, the tissue type restricted expression of *HES*, and the failure of other L18 members to complement the *hes* phenotype suggest that the L18 proteins are involved in modulating development. This is likely via heterogeneous mitoribosomes containing different L18 members, which may result in differential mitochondrial functions in response to different physiological situations during development.

## Highlight

An *Arabidopsis* L18 mitochondrial ribosomal protein modulates cell division, differentiation, and development, potentially with other diverse L18 family members, by forming heterogeneous ribosomes in mitochondria.

## Introduction

During plant development, cell division, growth, and differentiation are precisely coordinated and regulated to generate tissues that contribute to the formation of vegetative and reproductive organs. To understand the mechanisms controlling organ formation it is necessary to identify the underlying genes and endogenous processes. Proliferation and differentiation of cells and organ formation require energy and metabolites for the synthesis of proteins and other biomolecules to fuel or build these processes. *Arabidopsis* was used here to investigate the molecular mechanisms underlying early seed development.

Double fertilization, which initiates seed formation, occurs in the female gamete or embryo sac in the ovule of flowers and leads to the formation of the nutritive triploid endosperm and the diploid embryo, the progenitor of the seedling. Embryo development follows a series of stages identified as zygote, globular, heart, torpedo, and walking stick (Jurgens *et al.*, 1995). The basic plant body plan is established when the embryo reaches heart stage. The triploid endosperm progenitor nucleus undergoes nine rounds of division without cytokinesis, resulting in a multinucleate syncytium. This is followed by cellularization, and the endosperm is later consumed by the developing embryo (Sørensen *et al.*, 2002). The coordinated cell growth, differentiation, and patterning process of each component of the seed must be well regulated by genes and internal processes to form viable seeds. To date, over 400 independent embryo-lethal mutants, arrested at various stages of embryogenesis in *Arabidopsis*, have been generated in mutagenesis screens, providing a significant resource to investigate the molecular events underlying embryo and endosperm development (Meinke *et al.*, 2008). Characterization of some mutants has uncovered genes involved in the embryo patterning and endosperm cellularization. However, most of these are poorly characterized for specific molecular and developmental functions. A number of these mutations occur in ribosomal proteins involved in cytoplasmic protein synthesis (n = 7) or in ribosomal proteins targeted to mitochondria (n = 2) or plastids (n = 11), indicating the importance of mRNA translation in both the cytoplasm and in organelles for early seed development.

Mitochondria in eukaryotic cells evolved from ɑ-proteobacteria and are self-reproducing organelles where ATP is synthesized by respiration. Increasing evidence indicates that mitochondria have essential and multiple functions in cell proliferation, growth, cell differentiation, organ formation, and many other biological processes (McBride *et al.*, 2006; Maeda and Chida, 2013). The organelle has its own genome encoding over 50 genes, the transcripts of which are translated by mitochondrial ribosomes (mitoribosomes) (Unseld *et al.*, 1997). The making of functional mitochondria requires coordination between the organellar and nuclear genomes for transcription, post-transcriptional processing, translation, and post-translational processing of thousands of genes (Kwasniak *et al.*, 2013). The majority of mitochondrial proteins are encoded in the nuclear genome. Spatial and temporal expression patterns of these nuclear genes may influence the organelle function and subsequently plant development. A growing body of evidence shows that mutations in some nuclear genes encoding mitochondrially targeted proteins lead to specific developmental phenotypes, suggesting that this organelle may exert regulatory roles (Sakamoto *et al.*, 1996; Tsugeki *et al.*, 1996; Skinner *et al.*, 2001; Portereiko *et al.*, 2006; Van Aken *et al.*, 2007; Zhou et al 2011; Kwasniak *et al.*, 2013; Pineau *et al.*, 2013; Deng *et al.*, 2014; Pan *et al.*, 2014). Some of these mutations occur in the genes encoding mitoribosomal proteins. The observation that specific developmental phenotypes occur despite the presence of multiple genes encoding the same types of mitoribosomal proteins implies the formation of heterogeneous mitoribosomes, which may lead to selection or filtering of mitochondrial transcripts for translation to influence development, as suggested by the ribosome filter hypothesis (Mauro and Edelman, 2007; Janska and Kwasniak, 2014).

In this study, a screen was carried out for *Arabidopsis* mutants with seeds of altered phenotypes. A recessive mutant, *heart stopper* (*hes*), was found with reduced seed size that arrested at the late globular stage. *HEART STOPPER* is shown to encode a mitochondrion-localized L18 ribosomal (mitoribosomal) protein belonging to a small, ancient gene family that is widespread in higher plants. Characterization of this gene at molecular and morphological levels suggests that this gene plays a vital role in cell division and differentiation, and seed development.

## Materials and methods

### Mutant screening

*Arabidopsis* Landsberg *erecta* (L*er*) wild-type seeds were mutagenized with ethyl methanesulfonate as described by Chaudhury *et al.* (1997). In an M2 population consisting of about 10 000 plants, mutants showing various seed phenotypes were identified. Among them, a plant producing about 25% small developing seeds was isolated and these small seeds eventually aborted. The putative mutant was back-crossed to L*er* twice.

### Microscopy

The wild-type and mutant ovules were cleared as described by Garcia *et al.* (2003) and observed and captured as digital images using differential interference contrast microscopy with a Leica DMR compound microscope. Early seeds with GUS activity were observed and photographed.

Expression of green fluorescent protein (GFP) and MitoTracker Red was detected as described by Christensen *et al.*, (2002), with the roots mounted in 1× PBS solution. GFP images were captured using a Leica TCS SP2 confocal microscope.

For transmission electron microscopy (TEM), seeds were fixed and embedded according to Hyman and Jarvis (2011). Sections of ~90nm thickness were cut on a Reichert-Jung Ultracut E ultramicrotome and stained with 2% Uranyl acetate (Ajax Chemicals) for 10min and Reynolds lead citrate (BDH Ltd.) for 8min. The sections were viewed in a Hitachi H7000 transmission electron microscope at 75 KV. Images were taken using an SIA 12C digital camera.

### Embryo rescue

The embryo rescue procedure was as described by Sauer and Friml (2008). *hes* homozygote seeds (based on small seed size) 80–96 hours old were cultivated on *in vitro* culture of ovules media (ICM). Three-day-old wild-type seeds at the globular stage were used as controls. All the seeds were grown for 5 days on ICM then transferred to *Arabidopsis* medium (AM). Two weeks later they were transferred to Murashige and Skoog (MS) medium. Seed germination was scored.

Callus from the *hes* seeds on MS medium were transferred to a regeneration medium containing the hormones 1-naphthaleneacetic acid and 6-benzylaminopurine (Acedo 1986). Callus induced from wild-type seeds was treated similarly.

### Construction of the 3D molecular models of the HES proteins

3D models of the *Arabidopsis* HES proteins were constructed by comparative (homology) modelling that was based on special restraints of a suitable structural template (Sali and Blundell, 1993). The structural template for HES proteins was identified via 3D-PSSM (Kelley *et al.*, 2000), LOMETS (Wu and Zhang, 2007), MUSTER (Wu and Zhang, 2008), and the Structure Prediction Meta Server (Ginalski *et al.*, 2003). The most suitable template for modelling was found to be the L18 ribosomal protein from *Thermus thermophilus* (Woestenenk *et al.*, 2002) (PDB 1ILY:A). The 1ILY:A and the *Arabidopsis* HES protein sequences were analysed for the positions of secondary structures using PROMALS3D (Pei *et al.*, 2008) and for domain arrangements by ProDom (Corpet *et al.*, 1998) and SMART (Letunic *et al.*, 2012). The aligned 1ILY:A and HES sequences were used as input parameters to build 3D models on a Linux Red Hat workstation, running a Fedora Linux (release 12) operating system and the Modeller 9v8 program (Sali and Blundell, 1993). A final 3D molecular model of each HES protein was selected from 40 models that showed the lowest values of the ‘Modeller Objective Function’ and ‘Discrete Optimized Potential Energy’ parameters. The stereochemical quality and overall G-factors of the 1ILY:A structure and the HES models were calculated with PROCHECK (Laskowski *et al.*, 1993). Z-score values for combined energy profiles were evaluated by Prosa 2003 (Sippl, 1993). Structural superpositions of 1ILY:A (90 residues) and the HES models (96 residues) were performed using a PyMol (http://www.pymol.org accessed 11 June 2015) ‘superposition’ algorithm, where 88 residues excluding two insertions and one deletion were used. The electrostatic potentials were calculated with the Adaptive Poisson-Boltzmann Solver (the dielectric constants of solvent and solute were 80 and 2, respectively) (http://www.poissonboltzmann.org accessed 11 June 2015) implemented in PyMol as a plugin, and mapped on protein molecular surfaces that were generated with a probe radius of 1.4 Å. Molecular graphics was generated with a PyMol software package (http://www.pymol.org/ accessed 11 June 2015).

### Microarray analysis

Four different categories of developing seeds were isolated for microarray analysis in triplicate. All the seeds in the siliques of selfing wild-type and heterozygous *hes* plants were targeted at 60 hours after pollination (HAP), and normal and small sized seeds were separated from selfing *HES/hes* plants at 80 HAP. Immature seeds were isolated from multiple siliques using a dissecting microscope and transferred to a microfuge tube on dry ice. Total RNA was quantified using a Nanodrop spectrophotometer and RNA integrity was assessed from the nanofluidic electrophoresis trace generated using the BioRad Experion platform. For array hybridization, 500ng of total RNA was labelled by a single round of *in vitro* transcription using the Amino Allyl MessageAmp II aRNA kit (Ambion Inc.) following the manufacturer’s instructions for incorporation of amino allyl UTP. The amino allyl-modified aRNA was coupled to the NHS ester dye Cy5. Approximately 4 μg of dye-labelled antisense RNA per sample was used to hybridize the NimbleGen *Arabidopsis* 12×135K array on a NimbleGen Hybridization system following the manufacturer’s instructions. The new NimbleGen *Arabidopsis thaliana* 12×135K arrays were chosen because they give much better representation than the more established ATH_1 Affymetrix GeneChip. The NimbleGen probes are long oligos (60mers) and one array has four independent probes for each of the 39 042 target transcripts designed using the TAIR genome release 9.0. The slides were scanned using a NimbleGen MS 200 microarray scanner and images were processed and normalized (by Robust Multi-array Average) using the NimbleScan software.

For ontology analysis, genes that were present in 1.5-fold higher or lower quantities between treatments were identified using Microsoft Excel (Berardini *et al.*, 2004). These lists were uploaded to the VirtualPlant 1.0 website (http://virtualplant.bio.nyu.edu/cgi-bin/vpweb/ accessed 11 June 2015) and gene ontology (GO) analysis was carried out with the BioMaps function using TAIR ontology terms (*P*-value cut off of 0.05). To negate effects of GO bias inherent in the choice of the loci present on the array, a list of array-represented loci was used as the background reference list for GO comparisons.

Lists of mitochondria- and chloroplast-targeted gene products were obtained from http://suba.plantenergy.uwa.edu.au/ accessed 11 June 2015 (Heazlewood *et al.*, 2005). The mitochondrial stress regulon is described by Skirycz *et al.* (2010). These gene lists were cross referenced with the array data using Microsoft Access.

Further details on materials, growth conditions, genetic mapping, gene cloning, phylogenetic analysis, reporter gene constructs, and gene expression can be found in the Supplementary Methods at *JXB* online.

## Results

### Identification and characterization of the *hes* mutant

**Fig. 1.**
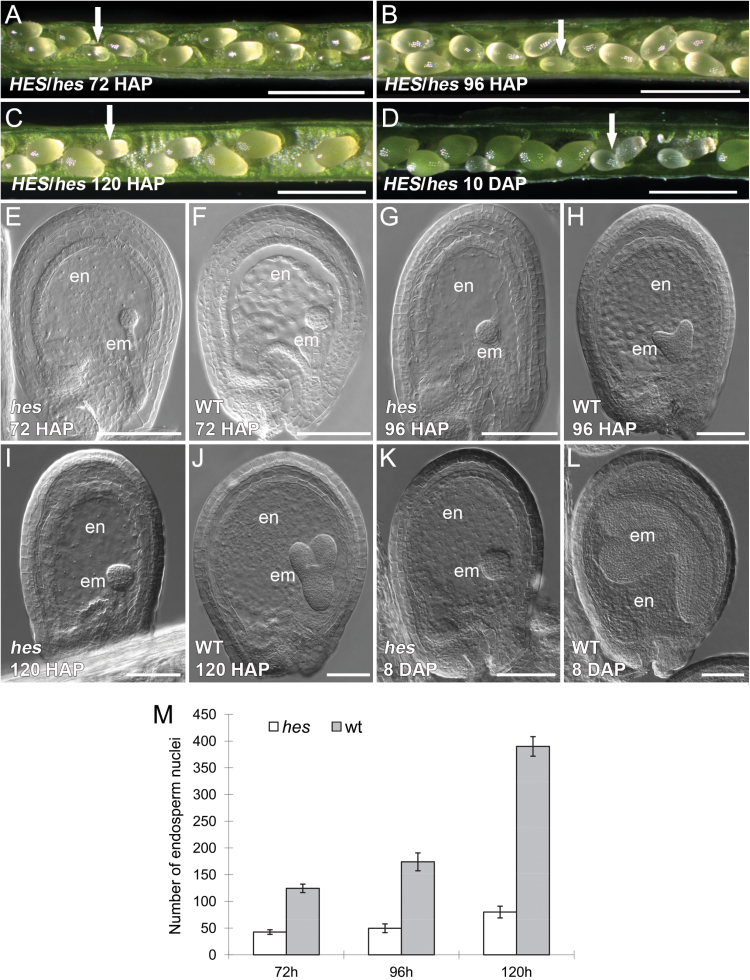
Characterization of the *hes* seed phenotype of heterozygous *HES/hes* plants. (A) Seeds in a silique, showing size difference between the *hes* (arrow indicating a smaller mutant seed) and wild-type seeds at 72 HAP. (B) Seeds in a silique at 96 HAP (arrow indicating a smaller mutant seed). (C) Seeds in a silique at 120 HAP (arrow indicating a smaller mutant seed). (D) Seeds in a silique ~10 DAP. The *hes* seeds remain small and abort. (E) A *hes* seed under differential interference contrast microscopy at 72 HAP. (F) A wild-type seed at 72 HAP. The *hes* seed has a smaller globular embryo compared to the wild type. (G) A *hes* seed at 96 HAP. The *hes* seed remains uncellularized with a globular embryo. (H) A wild-type seed at 96 HAP. The wild type starts cellularization with a heart stage embryo. (I) A *hes* seed at 120 HAP. (J) A wild-type seed at 120 HAP. (K) A *hes* seed at 8 DAP. (L) A wild-type seed at 8 DAP. (M) Numbers of endosperm nuclei in the *hes* and wild-type seeds at 72, 96 and 120 HAP. Scale bars: 1mm in A-D; 100 µm in E-L. em, embryo; en, endosperm; wt, wild type.

Ethyl methanesulfonate mutagenesis of the *Arabidopsis* L*er* ecotype resulted in an M2-generation plant exhibiting small seeds that had aborted in the early stages of seed development at silique maturity (Fig. 1A-D). There were about 25% aborted seeds (220 seeds scored) in the siliques of self-pollinated plants. When a *hes* heterozygote was used as pollen donor to a wild type, the progeny of resulting F1 seeds contained ~50% heterozygotes (37 wild type to 42 heterozygotes in the F2 population); similarly in the reciprocal cross, 50% heterozygotes were identified (27 wild type to 23 heterozygous). These results suggest that the mutation is strictly inherited in a sporophytic recessive manner. The mutation did not affect transmission via female and male gametophytes. Tests were then performed to see if there was any maternal gametophytic effect in early seeds possibly exerted by the *hes* mutation. When the heterozygous plants were pollinated with wild-type pollen, all the seeds developed normally. The heterozygous plants could not be distinguished from the wild type, suggesting no dominant negative effect exerted by the *hes* mutation.

Observations of seed development in opened siliques of the heterozygous mutant indicated uniform seed size until approximately 60 HAP. However, by 72 HAP, 28.4% of the seeds (n = 250) were smaller than the remaining seeds, and this trend continued in siliques observed at 96 and 120 HAP. At 10 days after pollination (DAP), the small putatively homozygous seeds remained white and some aborted (Fig. 1A-D). Images from differential interference contrast microscopy of both large and small seeds in siliques of the heterozygous mutant at 72, 96, and 120 HAP and also 8 DAP are shown in Fig. 1 (E-L). Embryo development in the smaller seeds progressed through early, mid, and late globular stages (Fig. 1E, G, I), and arrested at the late globular stage when the embryo had acquired a cubic appearance indicative of embarking on early transition to heart stage (Fig. 1K). In the larger seeds from the same siliques, all embryos progressed from mid-globular, heart, torpedo, and walking-stick stages (Fig. 1F, H, J, L), and seed development was identical to that of wild-type plants. In the small mutant seeds, endosperm development did not progress past the syncytial phase and cellularization was not observed (Fig. 1E, G, I, K). The endosperm division rate was slower in mutant seeds, producing lower numbers of endosperm nuclei (Fig. 1M). This mutant was named *heart stopper* (*hes*) because embryos arrested at the late globular stage entering the transition to heart stage.

The slow development of the endosperm suggests that the *hes* mutation might perturb the function of genes involved in endosperm proliferation. The *hes* mutant was crossed into three endosperm marker lines to investigate if *hes* altered expression of genes known to be involved in syncytial endosperm proliferation. The expression of MEA:GUS, FIS2:GUS (Luo *et al.*, 2000), and MINI3:GUS (Luo *et al.*, 2005) in the *hes* seeds was similar at 72 HAP when the wild-type embryo reached the mid globular stage (Supplementary Fig. S1A-L). At later stages, when wild type reached torpedo or late heart stage with cellularized endosperm and the GUS activity of the three markers had disappeared, the mutant seeds still displayed GUS activity, consistent with the observation that the *hes* endosperm could not reach cellularization (Supplementary Fig. S1A-L).

### Response of the *hes* embryo on culture media

**Fig. 2.**
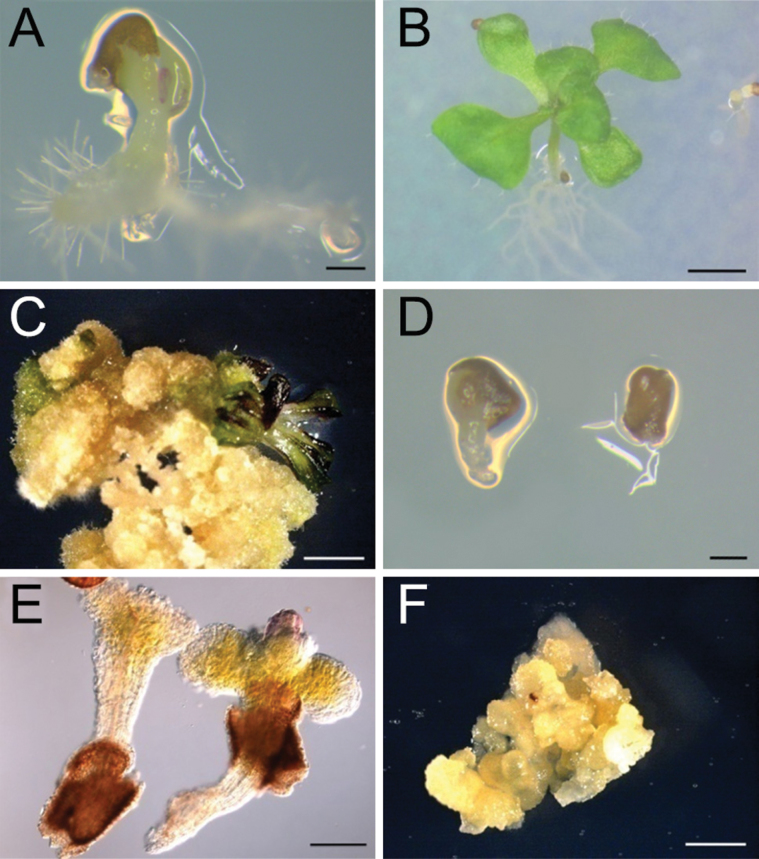
Rescuing the *hes* embryo using culture media. (A) A germinated wild-type seed on ICM medium. (B) A seedling from a rescued wild-type embryo. (C) Callus induced from wild-type embryo and producing shoot under induction. (D) *hes* seeds on ICM medium, showing one seed germinated. (E) *hes* embryos have developed to form elongated hypocotyls and cotyledons. One embryo has a bulb-like structure possibly derived from the apex. (F) Callus from the *hes* embryo. Scale bars: 200 µm in A and B; 2mm in C-F.

The arrested embryo phenotype in *hes* prompted an investigation of whether the embryos were viable and could be rescued in culture. Examination of the capacity for *hes* rescue and regeneration also provided an opportunity to examine roles of *HES* in promoting or regulating cellular differentiation. The embryo rescue technique of Sauer *et al.* (2008) enables the growth, differentiation, and germination of embryos from the 8-cell globular stage. Wild-type *Arabidopsis* seeds containing globular embryos (~72 HAP) and *hes* seeds containing embryos at the same globular stage of development (~80–96 HAP) were cultured on ICM (free of hormones) for 5 days, and subsequently on AM (containing hormones) for 2 weeks, and finally on MS medium for germination as described by Sauer *et al.* (2008). After 20 days in culture, ~30% of both wild type (n ≅ 200) and *hes* seeds (n ≅ 300) began to germinate on MS medium (Fig. 2A, D). Most of the germinated wild-type seeds developed into normal seedlings (Fig. 2B). By contrast, the *hes* seedlings were deformed with elongated hypocotyls, rudimentary cotyledon-like structures, and poorly developed roots (Fig. 2E). In half of these *hes* seedlings, a bulb-like structure developed in the position of the apical meristem (Fig. 2F). Approximately 30% of these *hes* seedlings developed into callus on MS medium, which is free of hormones (Fig. 2F). The origin of the callus from the *hes* embryo was confirmed by genotyping for the *hes* mutation (Supplementary Fig. S2B). The callus derived from the *hes* seeds could not be induced to differentiate into plants by adding hormones, in contrast to the callus derived from the wild-type seed, which underwent differentiation (Fig. 2C; Acedo, 1986). These results suggest that the *hes* embryos are viable when placed in culture but that the *hes* mutation has influenced further embryo differentiation, suggesting *HES* is required for cell growth and differentiation, and organ patterning.

### *HES* encodes an L18 ribosomal protein

Map-based cloning showed that the *hes* mutant contains a single nucleotide substitution of G to A at nucleotide 421 of the At1g08845 coding region, which is predicted to lead to a conserved glycine at position 141 being replaced by an arginine (Supplementary Figs S2, S3; Supplementary Methods). To further confirm that At1g08845 is the *HES* gene, a *HES/hes*-derived population containing heterozygote and wild type were transformed with a full-length genomic fragment of At1g08845. The plants carrying both the *hes* mutation and transgene show reduced seed abortion (Supplementary Methods; Supplementary Table S1). At1g08845 is predicted to encode an L18 ribosomal protein (www.arabidopsis.org accessed 11 June 2015).

### Expression of *HES* in plant tissues during development

The complete *HES* genomic sequence was fused at the C-terminus to the GUS colour imetric marker gene and was introduced into *Arabidopsis* to examine expression of the L18 gene during plant development. Five primary transgenic plants expressing the gene were examined and showed similar patterns of GUS staining. Developmental analyses of GUS expression and quantitative RT-PCR (qRT-PCR) analyses in different tissues were examined in plants homozygous for the transgene.

**Fig. 3.**
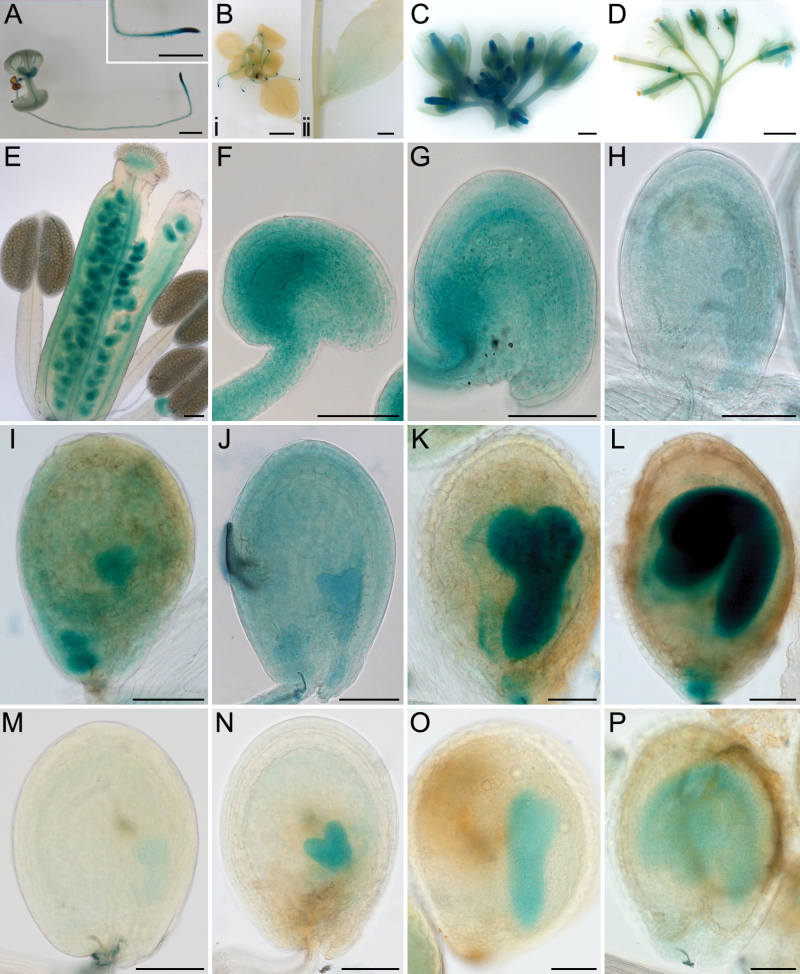
HES:GUS activity during plant development. Plant tissues were stained overnight unless otherwise indicated. (A) A 1-week-old seedling with GUS activity in the root tip, emerging leaves, and vein of cotyledons. Insert showing an enlarged root tip. (Bi) A 2-week-old seedling with GUS activity in the root tip (4h staining in A, Bi and M-P). (Bii) Stem and a cauline leaf. (C) GUS staining of upper part of the inflorescence showing early buds stained in blue. The petals, sepals, and anthers of older buds were negative for staining. (D) Lower part of the same inflorescence, showing siliques were negative for staining after development. (E) Closer image of a bud from C. (F) An unfertilized ovule just prior to pollination. (G) An early seed (~24 HAP). (H) A seed at mid globular stage. (I) A seed at late globular stage. (J) A seed at heart stage. (K) A seed at torpedo stage. (L) A seed at curled cotyledon stage. (M) A seed at late globular stage, showing GUS in the embryo and possibly in the endosperm. (N) A seed at mid heart stage, showing GUS in the embryo and weakly in the endosperm. (O) A seed at torpedo stage showing GUS activity in the embryo. (P) A seed with bent embryo ~8 day post fertilization. Scale bars: 1mm in A, C and D; 2mm in B; 100 µm in E-P.

Expression of the L18 gene was differentially regulated during plant development. Different levels of fusion protein expression as represented by the intensity of GUS staining and corresponding levels of transgene transcript were observed in different cell and tissue types by qRT-PCR (Fig. 3; Supplementary Fig. S4). In germinating seedlings and flowering plants, HES:GUS activity was detected preferentially in the root tips, emerging leaves, and veins of cotyledons (Fig. 3A, B), and GUS activity was absent in other parts of the plant or weakly positive after 4h of staining. After overnight staining, GUS activity was still negative in the stem but weakly positive in the cauline leaf (Fig. 3B). Expression was high in early developing flower buds, and was not observed at later stages in the anthers, petals, and sepals (Fig. 3C-E). Expression was high in the unfertilized ovule particularly in the chalazal region prior to pollination (Fig. 3F). Expression was also high post-fertilization in the maternal chalazal tissues of the ovule at ~24 HAP (Fig. 3G). Lower expression in the endosperm and embryo at mid globular stages was seen (Fig. 3H). GUS activity significantly increased in the embryo at late globular stage when the embryo was transitioning to heart stage and remained high in the embryo through embryogenesis until the curled cotyledon stage (Fig. 3I-L). With 4-h staining, the expression of L18 in the endosperm was weak at the early globular stages of development and remained low or was not detectable as embryogenesis occurred (Fig. 3M-P). The observed HES:GUS activity patterns during seed development are consistent with the timing of embryo and endosperm arrest in *hes* homozygous mutants.

### The evolution of L18 proteins and prediction of organelle targeting

**Fig. 4.**
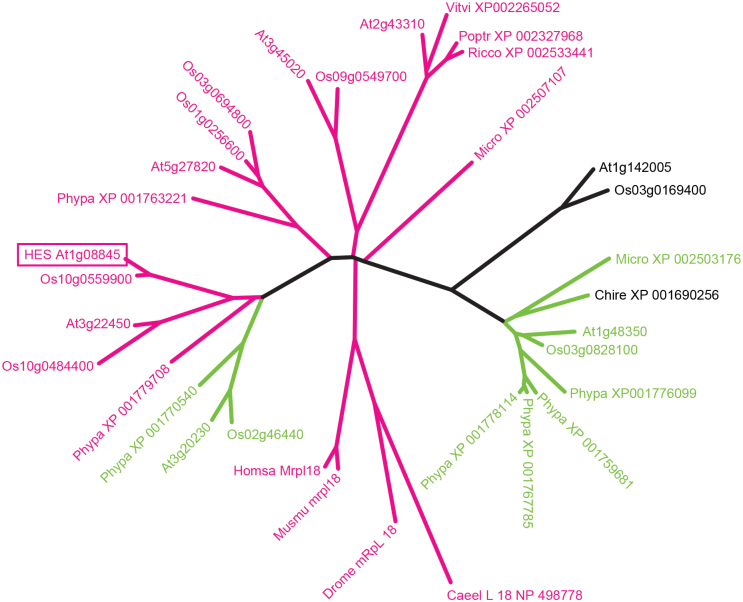
Phylogenetic tree of organelle ribosomal L18 protein sequence relationships. Higher plants are mostly represented by *Arabidopsis* (At) and *Oryza* (Os) sequences. The clade that includes At2g43310 protein is not present in monocots so more representative dicot proteins from *Vitis vinifera* (Vitvi), *Ricinus communis* (Ricco), and *Populus trichocarpa* (Portr) were included. Proteins from the moss *Physcomitrella patens (Phypa),* the unicellular Chlorophytan algae *Micromonas* (Micro), and *Chlamydomonas reinhardtii* (Chlre) as well as representatives of mitochondrial L18 proteins from the Metazoan organisms *Caenorhabditis elegans* (Caeel), *Drosophila melanogaster* (Droma), mouse (Musmu), and humans (Homsa) are also included. Sequence name are the respective Genome Initiative locus names or GenBank accession numbers. The alignment was made using ClustalW and the Neighbour Joining algorithm was used to construct the tree (green for putative chloroplast location and red for mitochondria). The ortholog of the At1g48350 protein is found in purified spinach chloroplast ribosomes as predicted from its transit peptide sequence.

PSI-blast searches were conducted using HES and related L18 sequences as queries across wide and restricted taxonomic ranges to identify the diverse L18 proteins in plants and to confirm single L18 genes/proteins in animals and other eukaryotes. These sequences are very diverse; for example, the HES L18 core sequence is only 19% identical to the NCBI L18 Conserved Domain consensus sequence. N-terminal sequences were interrogated using the Target P algorithm to predict the subcellular location (Emanuelsson *et al.*, 2000). The *Arabidopsis* genome codes for eight L18-like proteins, five of which are predicted to be transported to the mitochondria, two to the chloroplast, and one with an ambiguous targeting peptide which may lead to transport to either organelle. The amino acid sequence identities of HES to the different *Arabidopsis* L18 proteins ranged from 10% to 40%, while all proteins within the gymnosperm and angiosperm HES At1g08845 clade had >80% amino acid identity. A subset of these hits were used to construct the phylogenetic tree containing representative multicellular plant entries, as well as unicellular plants and four animal species (Fig. 4). This suggests that there are different classes of L18 that evolved before the divergence of higher plants and mosses.

### HES protein localizes to mitochondria

**Fig. 5.**
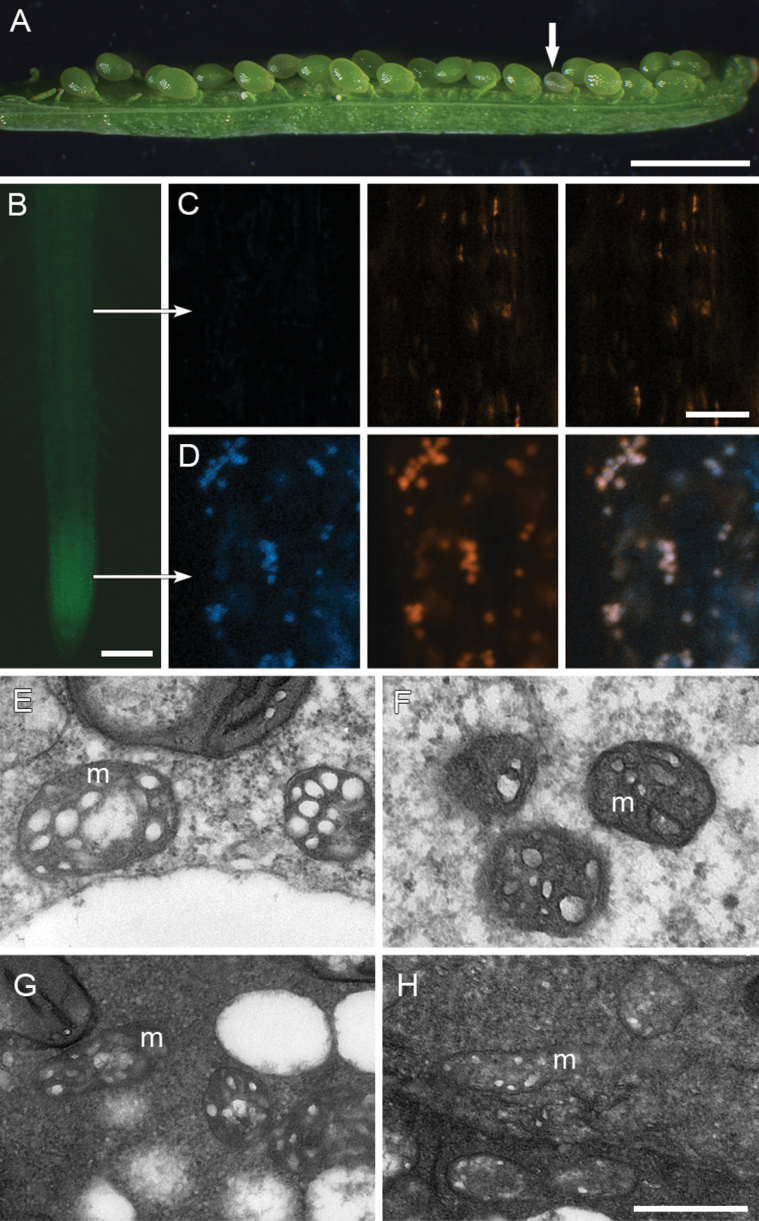
HES:GFP targets mitochondrion. (A) Silique of a *hes* heterozygous plant carrying HES:GFP, showing one small seed (arrow). (B) HES:GFP fluorescence in root tip. (C) Confocal microscope images in a cell from root mature region (upper arrow in B): left panel, negative HES:GFP fluorescence; middle panel, bodies stained with MitoTracker Deep Red; right panel, overlay. (D) Confocal microscope images in a cell from root tip (lower arrow in B): left panel, positive HES:GFP fluorescence; middle panel, bodies stained with MitoTracker Deep Red; right panel, overlay. (E-H) TEM images of mitochondria (m) from wild-type endosperm (E), *hes* endosperm (F), wild-type embryo (G) and *hes* embryo (H). Scale bars: 1.5mm (A); 100 μm (B); 500nm (E-H, bar shown in H), and 4 μm (C-D, bar shown in D).

The HES protein contains a predicted mitochondrial targeting peptide (Emanuelsson *et al.*, 2000) as expected of such L18 proteins that are found in organellar 70S ribosomes. To verify the subcellular location of HES, *HES/hes* heterozygote plants were transformed with a protein fusion construct in which the *HES* whole genomic sequence was fused at the C-terminus to a GFP coding sequence. Two *HES*/*hes* heterozygous plants carrying the *HES*:*GFP* constructs were identified that showed reduced frequencies of formation of aborted seeds (Fig. 5A; Supplementary Table S1), suggesting the seed lesion has been complemented by HES:GFP. HES:GFP was detected in multiple small intracellular compartments and co-localized with MitoTracker Red, a mitochondrion-specific dye in root tip cells but not detected in root mature cells (Fig. 5B-D), suggesting that the HES protein localized to the mitochondrion. Several cells (n = 7) were examined at the root tip where the GFP showed the highest activity, and all the cells had GFP with >85% (393/461) of the MitoTracker-stained bodies targeted by GFP. In contrast, all the cells (n = 4) in the mature region of roots show poor GFP activity with only ~7% (6/85) MitoTracker-positive bodies overlapping with the GFP signal.

To investigate if the *hes* mutation influences mitochondrion structure or morphology, TEM was conducted on the developing wild-type and *hes* seeds at ~96 HAP. This showed that the membranes were intact and the inner membrane was compartmented into cristae similarly in endosperm and embryo between both types of seeds, indicating that the *hes* mutation has no obvious effect on the mitochondrion morphology (Fig. 5E-H). The effects on development may therefore relate to disruptions in metabolic processes within the mitochondrion, requiring protein synthesis mediated by L18 function.

### 3D molecular models of HES mutant and wild-type proteins

**Fig. 6.**
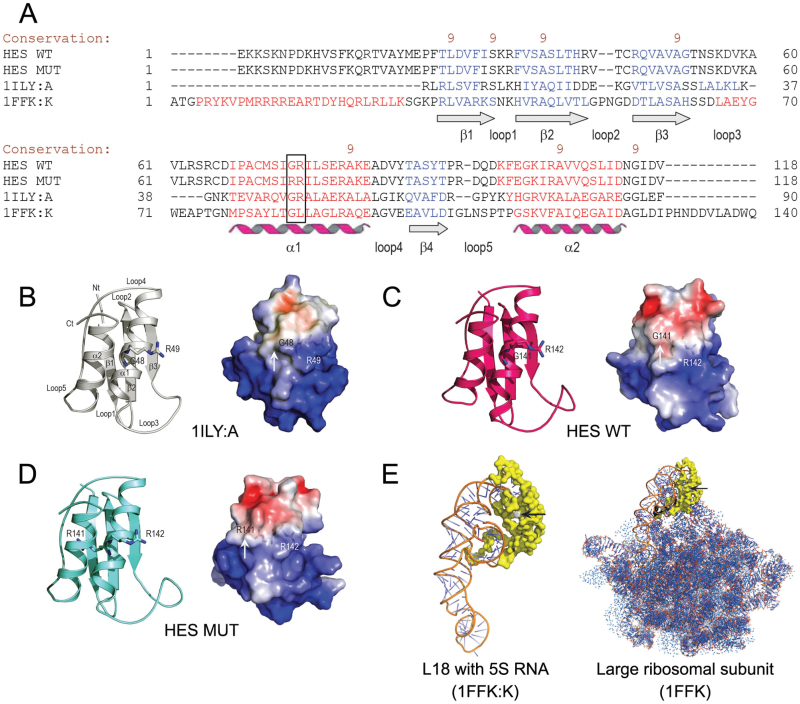
3D molecular models of HES proteins. (A) A sequence alignment of the *Arabidopsis* HES wild-type (WT) and G141R mutant (MUT) proteins, the L18 ribosomal protein from *T. thermophiles* (PDB accession 1ILY:A), and the L18 ribosomal protein from *H. marismortui* (PDB accession 1FFK:K) by PROMALS3D (Pei *et al.*, 2008). The black box indicates the GR/RR motif in the HES proteins and the corresponding regions in 1ILY:A and 1FKK:K. The degree of conservation of amino acid residues is shown by brown numbers on the scale 9-5. The secondary structures are indicated in blue (β-sheets 1–4) and red (α-helices 1 and 2). The adjoining loops are also indicated (cf. Fig. 6B). (B-D) 3D structure of the *T. thermophiles* L18 protein (B), and molecular models of the *Arabidopsis* HES wild-type (C) and G141R mutant proteins (D). The NH_2_-terminus (Nt) and COOH-terminus (Ct) are indicated alongside the secondary structural elements in the top left part of panel B. The G48 and R49, G141 and R142, and R141 and R142 residues in the 1ILY:A, and the HES wild-type, and mutant proteins are shown in sticks. The right panels illustrate molecular surface morphologies coded by electrostatic potentials. White, blue, and red patches on protein surfaces indicate electro-neutral, electropositive (5 kT/e), and electronegative patches (-5 kT/e) respectively. In the G141R mutant, an additional bulge is present on its surface (cf. white arrows in all three right panels). The residues of the GR and RR motifs in the wild-type and mutant proteins are illustrated by sticks and are visible under the molecular surfaces. (E) The 3D structure of the L18 ribosomal protein from *H. marismortui* (1FFK:K) with 5S rRNA bound (left panel). The right panel shows the 3D structure of the large (50S) ribosomal subunit (1FFK). The black arrows point to the GL region in 1FFK:K and 1FFK that corresponds to GR and RR regions in the *Arabidopsis* HES proteins (cf. Fig. 6A).

To explore how the glycine to arginine mutation might affect HES structure and mitoribosomal function, 3D molecular models of mutant and wild-type L18 proteins were constructed. The most appropriate template for generation of the HES protein structure was the *T. thermophilus* L18 protein [Protein Data Bank (PDB) 1ILY, chain A, designated 1ILY:A] (Woestenenk *et al.*, 2002). A total of 96 residues from full-length HES proteins (189 residues) were subjected to modelling. This 96-residue region spanning the mutation corresponded to a putative ribosomal domain L18 annotated by SMART (E-value of 3.10^−5^ against a Pfam database) (Finn *et al.*, 2010; Letunic *et al.*, 2012) and by ProDom (PD629301; E-value of 1e^−65^ against a ProDom database) (Corpet *et al.*, 1998). The sequence alignments of the L18 protein from *T. thermophilus* (Woestenenk *et al.*, 2002) with the HES wild-type and G141R mutant proteins showed 21% and 20% sequence identities, respectively, and 73% and 72% sequence similarities. Clearly, the two sequences fell within the so-called twilight type of modelling (Sanchez and Sali, 1998), although the structural superpositions showed similar dispositions of secondary structural elements aligned with PROMALS3D (Pei *et al.*, 2008) (Fig. 6A).

The secondary structural element dispositions and folds of the crystal structure of 1ILY:A and the HES models are shown in Fig. 6 (panels B-D). Superposition of the HES wild-type and G141R mutant proteins on the template crystal structure of 1ILY:A indicated that the respective structures matched closely, and had root-mean-square-deviation values of 0.50 Å and 0.58 Å for the Cα backbone positions in 88 structurally equivalent residues. The evaluations by PROCHECK (Laskowski *et al.*, 1993) and ProSa (Sippl, 1993) indicated that although the modelling of the HES proteins represented a twilight zone case, HES models have correct stereochemical parameters and thus can be considered reliable.

Owing to the glycine to arginine mutation, an additional bulge was present on the surface in the G141R mutant of the HES protein (Fig. 6D), although electrostatic potentials of all three proteins indicated similar charge distributions (Fig. 6, right panels in B-D). As illustrated in Fig. 6E, the L18 ribosomal protein from *Haloarcula marismortui* (PDB accession 1FFK:K) binds 5S rRNA directly. Thus, structural change of the G141R mutant HES protein that functionally corresponds to the L18 ribosomal protein could potentially lead to changes in binding of 5S rRNA and/or could influence the stability of the central protuberance of the 50S ribosomal subunit (Ben *et al.*, 2000), thus affecting the ribosome function in mitochondrial protein synthesis.

### Microarray analysis of the *hes* seeds

The effects on gene expression caused by the *hes* mutation were explored using microarray analyses to compare expression profiles in small seeds (homozygous for *hes*) and the larger seeds with wild-type–size phenotype harvested from siliques of the heterozygous *HES/hes* plants at 80 HAP. At this stage, the larger seeds were transitioning from the globular to the heart stage of embryo development. Seeds were collected from the *HES/hes* plants irrespective of the seed size and from wild-type plants at 60 HAP, as controls. Thus, transcriptome data was generated for four types of seed samples, each represented in triplicate on a single 12-array slide. At 60 HAP, 1608 genes were identified with a 2-fold difference in expression between seeds from *HES/hes* and wild type (Supplementary Table S2). Lipid transport was the only TAIR GO term (Berardini *et al.*, 2004) that was significantly enriched (*P* < 0.05) in wild type and was found in the list of genes that showed decreased expression in seeds from the *HES/hes* plants (Supplementary Table S3). This indicated that the molecular landscape of mutant and wild-type seeds in the 60 HAP samples was either very similar or that differences relating to the *hes* mutation were not readily apparent, because 75% of the seeds from the *HES/hes* siliques had normal development.

At 80 HAP, 2258 genes were differentially regulated between the *hes* seeds and the corresponding wild type-sized seeds (Supplementary Table S2). Twenty randomly selected genes with decreased expression in the *hes* seeds were verified by qRT-PCR (Supplementary Table S4). The GO analysis of genes up-regulated in the *hes* seeds indicated an over-representation of terms associated with a wide range of biological processes (Table 1; Supplementary Table S3). However, several of these ontology terms were similarly over-represented in comparisons between the wild-type seeds at 60 HAP and 80 HAP (Supplementary Table S3). To focus on genes directly affected by the *hes* mutation, the over-represented GO terms associated with developmental stage were removed. The GO terms not linked with developmental stage but significantly over-represented in the list of up-regulated genes in the *hes* seeds are shown in Table 1. These relate to aspects of salt/osmotic stress, jasmonic acid and abscisic acid signalling, flux in secondary metabolism, anion transport, and cytidine/pyrimidine metabolism.

**Table 1.** Representation analysis using TAIR ontology for up-regulated genes in the *hes* seed 80 HAP

Term^a^	Observed frequency (%)	Expected frequency (%)	*P*-value
response to osmotic stress	1.70	0.70	1.26E-05
response to salt stress	1.50	0.60	0.00029
response to jasmonic acid stimulus	1.20	0.50	0.00149
secondary metabolic process	2.30	1.30	0.00378
response to abiotic stimulus	4.50	3.10	0.00568
cytidine catabolic process	0.20	0.00	0.00716
nucleoside catabolic process	0.20	0.00	0.00716
cytidine deamination	0.20	0.00	0.00716
ribonucleoside catabolic process	0.20	0.00	0.00716
cytidine metabolic process	0.20	0.00	0.00716
pyrimidine ribonucleoside catabolic process	0.20	0.00	0.00716
pyrimidine nucleoside catabolic process	0.20	0.00	0.00716
inorganic anion transport	0.50	0.10	0.01377
anion transport	0.60	0.20	0.01931
pyrimidine ribonucleoside metabolic process	0.20	0.00	0.01981
pyrimidine nucleoside metabolic process	0.20	0.00	0.04563
abscisic acid mediated signalling	0.60	0.20	0.04738

^a^ Over-represented groups associated with developmental stages were removed (see Supplementary Table S5 for complete list).

Next, some of the known genes related to dysfunctional mitochondria were analysed. Twenty three genes have been used as markers for mitochondrial dysfunction and termed the mitochondrial dysfunction regulon (Van Aken *et al.*, 2007; Skirycz *et al.*, 2010) (Supplementary Table S5) and 13 of these genes are up-regulated in proliferating cells undergoing osmotic stress (Skirycz *et al.*, 2010). Similarly, 12 genes were up-regulated in the *hes* seeds, including *ALTERNATIVE OXIDASE 1a* (*AOX1a*, AT3G22370), *NAD(P)H* dehydrogenase gene (*NDB4,* At2g20800), and *UP-REGULATED BY OXIDATIVE STRESS* (*UPOX*, AT2G21640) (Vanlerberghe and McIntosh 1997; Gadjev *et al.*, 2006; Smith *et al.*, 2011) (Supplementary Table S5). Using qRT-PCR on independent samples, up-regulation of these 12 genes was confirmed (Supplementary Table S5) as well as of an additional *PROHIBITIN* gene, *ATPHB7* (At5g44140), which is from a gene family with members associated with mitochondrial dysfunction in *Drosophila* (Supplementary Table S6). Other members of the *NDB* and *AOX1* families are not significantly changed in the conditions examined.

A similar process was carried out for genes down-regulated in the *hes* seeds but no common GO terms were observed in a comparison of the mutant and wild-type and the different developmental stages (Supplementary Table S3). The *hes* phenotype at 80 HAP was associated with a slight down-regulation of a number of genes associated with the cell cycle, including three genes down-regulated in roots of the mitochondrially dysfunctional *rrg* mutant (Zhou *et al.*, 2011; Supplementary Tables S3 and S7). Furthermore, genes associated with mutants showing aberrant endosperm development exhibited very little differential expression in the *hes* seeds versus wild type at 80 HAP (Supplementary Table S8). Interestingly, GO analysis showed a significant number of photosynthesis genes (Supplementary Tables S3 and S9) were down-regulated in the *hes* seeds. Because plastids start to differentiate into chloroplasts at globular stage, this observation may indicate that mitochondria, directly or indirectly, play a role in plastid differentiation. A further ~400 *EMBRYO DEFECTIVE* genes of diverse function were examined in the *hes* seeds; 16 genes were down-regulated (Meinke *et al.*, 2008; Supplementary Table S10), of which six was also down-regulated in wild type at 60 HAP compared with at 80 HAP. This suggests that the regulation of many aspects of embryo and endosperm development can be modulated by mitochondrial function via the *EMBRYO DEFECTIVE* genes, and many other genes showing altered expression.

Translation of mitochondrial transcripts and expression levels of mitochondrial rRNAs can be differentially affected by alterations in mitochondrial ribosomes mediated by silencing nuclear *RPS10*, which encodes the S10 protein of the small subunit of the mitoribosome (Kwasniak *et al.*, 2013). The levels of mitochondrial transcripts were further examined, including microarray data for rRNA 5s, 18s, and 26s in the *hes* and wild-type–sized seeds. No evidence was found to suggest that the *hes* mutation influences transcription in mitochondria (Supplementary Table S2 column A: genes ATMG00010–ATMG01410).

## Discussion

A recessive *Arabidopsis* mutant, *hes*, that displays reduced endosperm proliferation and shows arrested embryos at the late globular stage has here been identified. Nuclear *HES* (At1g08845) encodes a mitochondrial protein homologous to the L18 ribosomal protein of eubacteria, which is a component of the central protuberance of the 70S ribosome’s large (50S) subunit and one of the two ribosomal proteins that binds to the 5S and 23S rRNA and is essential for protein translation (Shajani *et al.*, 2011). A multidisciplinary characterization of the *hes* mutant suggests that L18 is essential for mitoribosomal function and plays important roles in modulating cell division, differentiation, and early seed development.

### The *hes* mutation causes mitochondrial dysfunction via disruption of the mitochondrial translation machinery and activates a non-phosphorylating alternative oxidase pathway

Mitochondria need functional mitoribosomes to translate over 50 protein-coding transcripts encoded in the 367kb mitochondrial genome, including seven mitoribosomal proteins and most subunits of the major non-photosynthetic energy transducing complexes of the plant (Unseld *et al.*, 1997). 3D modelling suggests that a structural change due to a highly conserved internal glycine being substituted with the bulkier arginine in the hes L18 mitoribosomal protein could potentially lead to altered binding of 5S rRNA and/or other proteins of the central protuberance, L5 and L25. The hes mutant protein may be unstable and readily degraded or it may be incorporated into a mitoribosome, resulting in an inactive mitoribosome and thus damaging mitochondrial function. Unlike mutations in other mitochondrion-targeted proteins, which cause abnormal structures of mitochondria (Zhou *et al.*, 2011; Pineau *et al.*, 2013), no obvious structural change was found in the mitochondria of either the embryo or endosperm of the *hes* seed, suggesting that only the metabolic processes within the mitochondria were disrupted by the L18 mutation.

Dysfunctional mitochondria lead to the accumulation of reduced electron transport intermediates, increased reactive oxygen species (ROS) production, and a deficiency of ATP to deal with normal metabolism (Vanlerberghe and McIntosh 1997; Gadjev *et al.*, 2006; Millar *et al.*, 2011; Smith *et al.*, 2011). Gene expression profiling indicated that the developing *hes* seed responds to these deficiencies, notably by up-regulating expression of the nuclear-encoded non-phosphorylating alternative oxidase pathway, but, of course, cannot correct such a fundamental deficiency caused by the loss of mitoribosomal function. The increased expression (~13- and ~70-fold, respectively) of two genes in the so-called mitochondrial dysfunction regulon points to a specific and explicable molecular response to a growing loss of mitochondrial function. These genes encode two sequential enzymes in the non-phosphorylating alternative oxidase pathway, an external NAD(P)H dehydrogenase (NDB4) and an alternative oxidase (AOX1a) (Smith *et al.*, 2011). This response could provide a means to lower the high levels of cytosolic reduced NAD(P)H that will build up in the absence of sufficient mitochondrial respiratory electron transport. Unlike the phosphorylating pathway, the genes encoding the non-phosphorylating pathway are all nuclear and thus independent of a functional mitochondrial translation apparatus. Failure of mitochondrial energy transduction can produce elevated ROS (Millar *et al.*, 2011), which in turn, in the absence of sufficient ATP for cell maintenance, further damages mitochondria, producing more ROS. Thus, the up-regulation of genes in the mitochondrial dysfunction regulon, including *NDB4* and *AOX1a*, is likely a response to overproduction of ROS (Vanlerberghe and McIntosh 1997; Gadjev *et al.*, 2006; Busi *et al.*, 2011; Skirycz *et al.*, 2010).

### The mitoribosomal L18 protein HES is required for cell division and differentiation, and organ patterning

The up- and down-regulated genes in *hes* seeds have diverse functions, consistent with the multiple roles of mitochondria in a wide range of biological processes (McBride *et al.*, 2006; Maeda and Chida, 2013; Schwärzlander and Finkemeier, 2013). Reports have shown that the function of nuclear genes encoding mitochondrial-targeted proteins is required for cell division in roots and other aspects of plant development (Sakamoto *et al.*, 1996; Tsugeki *et al.*, 1996; Skinner *et al.*, 2001; Portereiko *et al.*, 2006; Van Aken *et al.*, 2007; Zhou *et al.*, 2011; Kwasniak *et al.*, 2013; Pineau *et al.*, 2013; Deng *et al.*, 2014; Pan *et al.*, 2014). The slow growth of both embryo and endosperm, and the interruption of the transition from globular embryo to heart stage and syncytial endosperm to cellularized endosperm in *hes* seed suggest that the L18 HES protein is indispensable for the cell division and differentiation which underlie early embryo and endosperm development. The slow growth of both fertilization products indicates a cell cycle progression defect and may be directly related to the down-regulation of cell cycle genes, three of which were also shown to be down-regulated in mitochondria-compromised roots (Zhou *et al.*, 2011). The results are also consistent with the analysis of cell cycle functions in seed development and meristem integrity, where altering expression levels of *CYCLIN* genes or *CDK* genes impacts on cell division and differentiation (Andersen *et al.*, 2008; Collins *et al.*, 2012).

Analysis of the *hes* phenotypes also revealed a novel role of mitochondria in the endosperm cellularization. As the expression of genes participating in cellularization was not affected in the *hes* seed, the mitochondrial function in this process may be involved in other pathways that modulate cellularization. There is evidence that sugar may act as signal for endosperm cellularization (Ohto *et al.*, 2005). Because sugar is a substrate for respiration, it is plausible that cellularization mediated by sugar signalling depends on mitochondrial function.

Experiments on how the *hes* embryo responds to rescue on culture medium provide further evidence about the role of mitochondria in cell division and differentiation, and organ patterning. The exogenous sugar in the medium might serve as a substrate for the compromised mitochondria to produce ATP for the arrested embryo, partially restoring cell division, growth, and even some differentiation (the formation of cotyledon-like and primary root-like structures). However, the abnormal seedlings from the rescued embryos were retarded and about 30% of them produced callus derived from the apex, suggesting that application of exogenous sugar might fuel a few rounds of slow cell division, producing seedling-like structures, but that those seedlings fail to further differentiate and form proper organs. In the cellular slime mould *Dictyostelium*, the expression of the mitoribosomal protein S4 gene is required for the initial differentiation (Maeda and Chida, 2013), indicating direct roles of mitoribosomes in differentiation. Observations from the present study are also consistent with the recent discovery that meristem activation and root growth driven by the glucose-TOR signalling require functional mitochondria (Xiong *et al.*, 2013). Further analysis indicated that 16 *EMBRYO DEFECTIVE* genes involved in multiple aspects of embryo and endosperm development (Meinke, 1992; Lukowitz *et al.*, 1996; Kondou *et al.*, 2008; Meinke *et al.*, 2008; Bayer *et al.*, 2009) are also down-regulated in *hes* seed. This suggests that mitochondrial function is linked by these genes, and many other genes showing altered expression, to the regulation of many processes of embryo and endosperm development. A recent analysis of glucose-TOR signalling in roots and other evidence support the idea that mitochondria are an integral part of multiple cell signalling cascades required for normal cellular function (McBride *et al.*, 2006; Schwarzländer and Finkemeier, 2013; Xiong *et al.*, 2013). It is suggested here that dysfunctional mitochondria caused by loss of the HES function may directly perturb the multiple signalling cascades as well as the provision of cellular ATP, which is essential for cell division and differentiation, and organ patterning.

### The tissue-type restricted expression, divergence of L18 family, and the failure of other L18 family members to complement the *hes* phenotype suggest the formation of heterogeneous ribosomes that may have specific functions in development

The *HES* gene is developmentally regulated, being preferentially expressed in tissues where there is active cell division and differentiation, including the developing embryo and the root tip. The early patterning of *hes* embryos appeared normal but the embryo arrested at late globular stage. There was no HES:GUS activity observed in the early embryo but strong activity occurred in the late globular embryo, suggesting that HES plays a critical role at the late globular stage. Therefore, other L18 proteins may function to support development of the early embryo. It is also apparent that *HES* is required for the division of syncytial endosperm and cellularization. Presumably the *hes* embryo and endosperm mutant phenotype is manifest because of the failure of the other L18 proteins to compensate for the absence of HES. The *hes* gene is transmitted efficiently in both gametophytic stages so it is also likely that the other L18 gene family members are active during these stages, implying that different L18 proteins may function in different tissues. The L18 gene families are highly diverged in sequence and evolutionary time, suggesting that the individual members may have slightly different properties and that the ribosomes containing them may also have different properties or specificities. The developmentally regulated expression of the *HES* gene suggests that different types of L18 are made and incorporated into mitoribosomes in a temporal and spatial manner, which results in the formation of heterogeneous mitoribosomes. This is consistent with the observation that most of the mitochondria at the root tip are targeted by HES:GFP, suggesting that the L18 encoded by *HES* is likely predominant over other L18 proteins in the mitoribosome assembled in the tissues where *HES* is highly expressed. It is worth mentioning that in addition to L18 other 15 mitoribosomal proteins are encoded by small or multi-gene families, indicating the complex formation of heterogeneous mitoribosomes (Sormani *et al.*, 2011). Recent results show that mitoribosomes can regulate gene expression by varying the efficiency of translation of mRNAs for oxidative phosphorylation system and ribosomal proteins (Kwasniak *et al.*, 2013; Janska and Kwasniak 2014). Therefore, heterogeneous mitoribosomes may exert specific functions by differentially selecting or filtering mRNAs for translation in response to fundamentally different physiological situations during development. The divergence of the L18 family, the tissue-type restricted expression of *HES*, and the failure of other L18 family members to complement the *hes* phenotype imply that the heterogeneous ribosomes comprising different L18 members may result in differential mitochondrial protein syntheses between tissues, and subsequently influence organ patterning and development. Consistent with this, development-specific phenotypes were observed in mutants defective in other mitoribosomal proteins (Sakamoto *et al.*, 1996; Tsugeki *et al.*, 1996; Skinner *et al.*, 2001; Portereiko *et al.*, 2006; Kwasniak *et al.*, 2013). Moreover, small families of cytosolic ribosomal proteins are also under developmental control and their mutants display tissue-specific phenotypes (Byrne, 2009). Together, these results support the ribosome filter hypothesis that ribosomes are not simply translation machines but also function as regulatory elements that select mRNAs for translation (Mauro and Edelman, 2007). The L18 family in plants is extreme in this regard, with eight members in *Arabidopsis*, and this diversity has been maintained for hundreds of millions of years. A proteomic analysis will be helpful to understand how the HES and other mitochondrial L18 proteins exert different functions in different tissues and how the *hes* mutation perturbs the translation in mitochondria.

## Supplementary material

Supplementary material is available at *JXB* online.

Supplementary Methods. Materials, growth conditions, genetic mapping, gene cloning, phylogenetic analysis, reporter gene constructs, and gene expression.

Supplementary Fig. S1. Expression of several endosperm and embryo markers in the *hes* and wild-type seeds.

Supplementary Fig. S2. Map-based cloning of the *HES* gene.

Supplementary Fig. S3. Cluster alignment of amino acid sequences of putative plant HES orthologues.

Supplementary Fig. S4. Quantitative PCR with the *HES* gene in various plant tissues.

Supplementary Table S1. Complementation of the *hes* phenotype by introducing At1g08845 genomic sequence into a population derived from *HES/hes.*

Supplementary Table S2. Gene expression in four seed samples.

Supplementary Table S3. GO analysis of genes up- and down-regulated in wild-type seeds 60 HAP compared to wild-type seeds 80 HAP.

Supplementary Table S4. Q-PCR verification of several down-regulated genes in the *hes* seeds.

Supplementary Table S5. Fold change of the mitochondrial dysfunction regulon genes between the *hes* and wild-type seeds.

Supplementary Table S6. Fold change of genes targeting mitochondria between the *hes* and wild-type seeds.

Supplementary Table S7. Fold change of selected cell cycle genes between seed samples.

Supplementary Table S8. Fold change of selected genes involved in endosperm development between seed samples.

Supplementary Table S9. Fold change of genes targeting chloroplast between the *hes* and wild-type seeds.

Supplementary Table S10. Expression of *EMBRYO DEFFECTIVE* genes in the *hes* seed.

Supplementary Table S11. Sequences and genetic distances to *HES* of PCR based mapping markers.

Supplementary Data
