# Supplementary material for: Developmentally regulated HEART STOPPER, a mitochondrially targeted L18 ribosomal protein gene, is required for cell division, differentiation, and seed development in Arabidopsis
Source: J Exp Bot. 2015 Jun 23;66(19):5867–80. doi: 10.1093/jxb/erv296 (PMC4566979; doi:10.1093/jxb/erv296)
Supplement: Supplementary Data [file supp_66_19_5867__index.html]

Developmentally regulated HEART STOPPER, a mitochondrially targeted L18 ribosomal protein gene, is required for cell division, differentiation, and seed development in Arabidopsis — Supplementary Data 

# Developmentally regulated *HEART STOPPER*, a mitochondrially targeted L18 ribosomal protein gene, is required for cell division, differentiation, and seed development in *Arabidopsis*

## Supplementary Data

Data files

- Supplementary Data - Supplementary Data
- Supplementary Data - Supplementary Data
- Supplementary Data - Supplementary Data
