# Supplementary material for: Developmentally regulated HEART STOPPER, a mitochondrially targeted L18 ribosomal protein gene, is required for cell division, differentiation, and seed development in Arabidopsis
Source: J Exp Bot. 2015 Jun 23;66(19):5867–80. doi: 10.1093/jxb/erv296 (PMC4566979; doi:10.1093/jxb/erv296)
Supplement: Supplementary Data [file supp_erv296_JEXPBOT_145201_supplementary_figures.pdf]

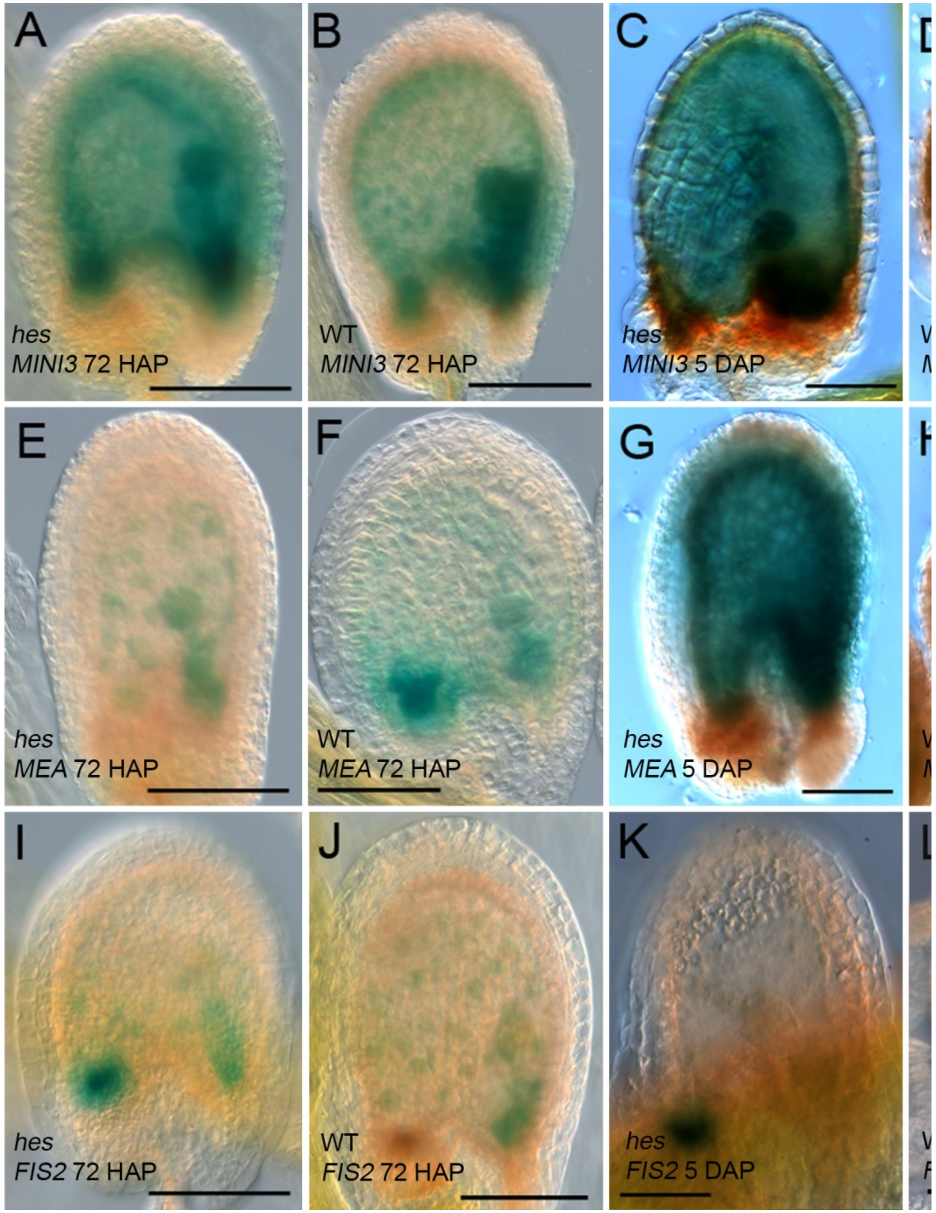

Fig. S1. Expression of several endosperm and embryo markers in the *hes* and wild type seeds.

(A and B) MINI3:GUS in a *hes* (A) and wild type (B) seed at 72 HAP.

(C and D) GUS seen in a *hes* seed (C) but not in a wild type seed (D) at 5 days after pollination (DAP).

(E and F) MEA:GUS in a *hes* (E) and wild type (F) seed at 72 HAP.

(G and H) GUS seen in a *hes* seed (G) but not in a wild type seed (H) at 5 DAP.

(I and J) FIS2:GUS in a *hes* (I) and wild type (J) seed at 72 HAP,.

(K and L) GUS seen in a *hes* seed (K) but not in a wild type seed (L) at 5 DAP. Scale bars: 100 µm in A-L.

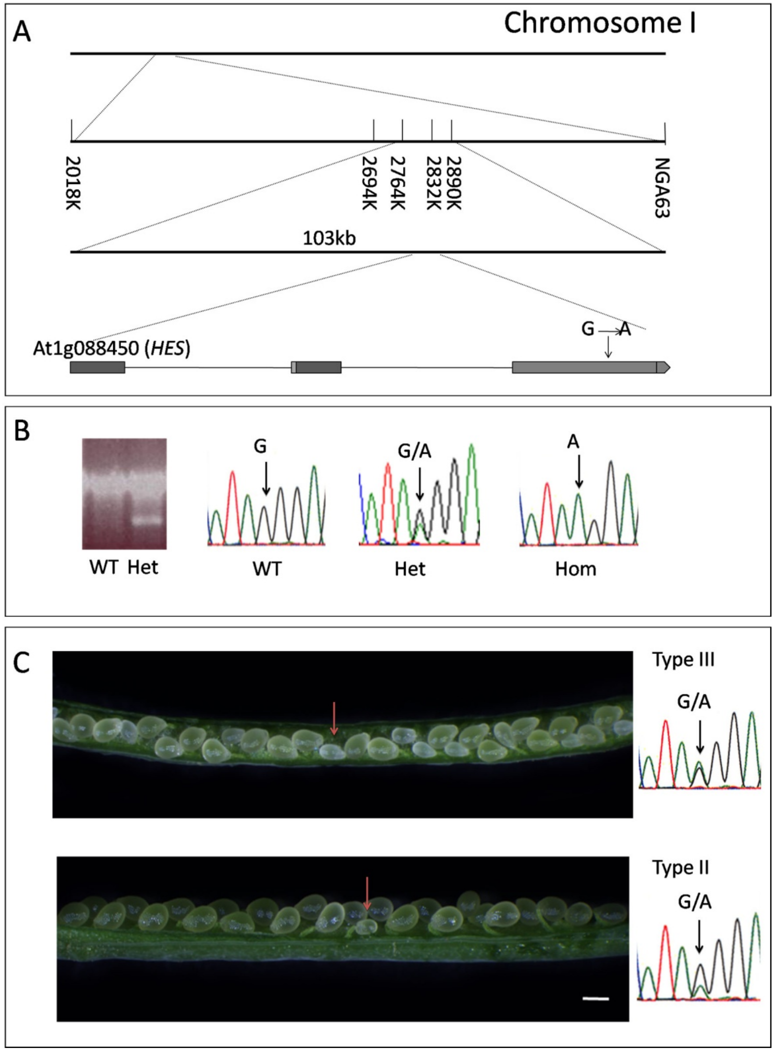

Fig. S2. Map based cloning of the *HES* gene.

(A) Detailed location of *HES*. *HES* is mapped in a region of 103 kb between marker 2764K and 2867K (Table S11).

(B) A mutation detected in At1g08845 in *hes*. The agarose gel showing that a PCR fragment of At1g08845 from wild type could not be cut but PCR fragment from a *hes* heterozygous plant could be cut with CEL1. Sequencing chromatographs showing G in wild type, G/A in heterozygote, and A in the *hes* homozygous callus.

(C) Complementation of *hes* with At1g08845 genomic fragment. Upper panel showing a silique with normal segregating of small white seeds and green normal seeds in a type III transgenic plant (Table S1), accompanying a sequencing trace showing a heterozygous A/G. All the seedlings derived from this plant are Kanamycin resistant. Lower panel showing a silique with one small seed in a type II transgenic plant (Table S1), accompanying a sequencing trace showing a heterozygous A/G. Scale bar: 400 µm in C.

```
                      *        20         *        40         *        60         *        80         *       100         *       120
Aegta     : --MLKNVLSQSWRRG--AHVLREGHPAEAMYACSSQFHSGQVLRSARSFFGVEDFMDEDNSKPYTYKKEKRSKNPH-KHISFKQRTIAHMEPFTLDVFISKRFVSASLTHRSTCRQVAVAGTNS : 119
Araly     : --MLKQVVTKYSIGKAAIYGSNREKLRPFLCQTSCRFHNARTVFAPRSFFGVEDYVDDDTSRPYTYQKEKKSKNPD-KHVSFKQRTVAYMEPFTLDVFISKRFVSASLTHRVTCRQVAVAGTNS : 121
Arath_HES : --MLKQVVAKYSIGKAAIYGSNREKLRPFMCQTSCRFHNGRTVFAPRSFFGVEDYVDDDTSRPYTYQKEKKSKNPD-KHVSFKQRTVAYMEPFTLDVFISKRFVSASLTHRVTCRQVAVAGTNS : 121
Arahy     : MNMLKQAVGRIWKSSSGYALEGGTKILAWSYVPSIYFHNTQAHTAPRSFFGVEDFLDDDNSKPYTYQKGKKSKNPH-KHVSFKQRTVAYMEPFTLDVFISKRFVSASLTHRVTSKQVAVSGTNS : 123
Aspof     : --MLRPVWRS-------WRNNAQNRSLPSFVQRTCGFHCGQVLFSPRSFFGVEDFLDDDNSRPYTYQKGKKSKNPQ-KHISFKQRTIAFMEPFSLDVFISKRFVSASLTHRVTCKQVAVAGTNS : 114
Bradi     : --MLKNIFSQSWRRG--AHVLQEGHPTAALYTCSSRFHGGQALCSSRSFFGVEDFMDEDNSRPYTYKKEKRSKNPH-KHISFKQRTIAHMEPFTLDVFISKRFVSASLTHRSTCRQVAVAGTNS : 119
Brana     : --MLKQVVG---IGK-AIYGSNRVKLRPFLCQTSCGFHNGRTVLAPRSFFGVEDYVDDDTSRPYTYQKEKKSKNPD-KHVSFKQRTVAYMEPFTLDVFISKRFVSASLTHRVTCKQVAVAGTNS : 117
Braol     : --MLKQVVG---IGK-AIYGSNRAKLRPFLCQTSCGFHNGRTVLAPRSFFGVEDYVDDDTSRPYTYQKEKKSKNPD-KHVSFKQRTVAYMEPFTLDVFISKRFVSASLTHRVTCKQVAVAGTNS : 117
Cenma     : --MLKRVCSMS------YTHGSEKTKLCFQ-AITRHFHSGQPHSAARSFFGVEDFLDDDNSRPYTYQKEKKSKNPN-KHISFKQRTIAYMEPFTLDVFISRRFVSASLTHRVTSKQVAVAGTNS : 114
Cicar     : --MLKQAIGRICRAN-VYSCEG-NRVLPWFYVPSCFFHNGQVHNAPRSFFGVEDFIDDDNSRPYTYQKGKKSKNPN-KHISFKQRTVAYMEPFTLDVFISKRFVSAALTHRVTSKQVAVAGTNS : 119
Cicen     : --MLKRICSMS------YEHGGETTKLCLQ-AITRHIHSGQARSSARSFFGVEDFLDDDNSRPYTYQKEKKSKNPN-KHISFKQRTIAYMEPFTLDVFISKRFVSASLTHRVTSRQVAVAGTNS : 114
Citcl     : --MLKQVVGKVLNNW-LVANGGEKAIRPFLYVPTSSLHQGQVLNAPRSFFGVEDFLDDDNSRPYTYKKEKKSKNTT-KHISFKQRTVAYMEPFTLDVFISKRFVSASLTHRVTSKQVAVAGTNS : 120
Cofca     : ------------------------SRSILH---PLYNFILQISFAPRSFFGVEDFLDDDNSRPYTYQKEKKTKNPN-KHISFKQRTIAYMEPFTLDVLISKRFVSASITHRVTSKQVAVAGTNS :  96
Cycru     : --MFKQLLGDVCNKG---KNTLSIIHLQVSSIFAREIHGGKGVCAPRSFFGVEDFVDEDNTRPYTYRKEKTSKNPK-KHVSFKQRTIAYMEPFTLDVFISKRFVSASITHRVTSRQVAVAGTNS : 118
Elagu     : --MFKNIFSNTRRN---VFFVPEYRKRAVSYVFRNNFHDGQVLSAPRSFFGVEDFVDDDNSRPYTYKKEKKSKNPQ-KHVSFKQRTIAYMEPFSLDVFISKRFVSASLTHRVTCKQVAVAGTNS : 118
Eutsa     : --MLKQVVGKYSIGK-AIYGSNRAKLRPFLCQTNCGFHNGRTALAPRSFFGVEDYVDDDTSRPYTYQKEKKSKNPD-KHVSFKQRTVAYMEPFTLDVFISKLFVSASLTHRVTCKQVAVAGTNS : 120
Glyma     : --MLKQAIGRIFRTG-VYACEG-NRVLPWIYVPSSFFHHGQAHMAPRSFFGVEDFLDDDNSRPYTYQKGKKSKNPN-KHISFKQRTIAYMEPFTLDVFISKRFVSASLTHRVTSKQVAVAGTNS : 119
Gosra     : --MLKQAIWKVLSKR-VVSESGDKLLLPCLYLPTTSFHAGQVHCSPRSFFGVEDFLDDDNSKPYTYQKGKKSKNPN-KHVSFKQRTEAYMEPFTLDVFISKRFVSASVTHRVTCKQVAVAGTNS : 120
Helan     : --MSAAFFRLS------AALSALFARPQPAGRLLRSSLSTQACFVPRSFFGVEDFLDDDNSRPYTYQKEKKSKNPN-KHISFKQRTIAYMEPFTLDVFISKRFVSASLTHRVTSKQVAVAGTNS : 115
Helci     : --MFKRACSM---------HGDDTAKLCLK-AIARHIHSAQARFGPRSFFGVEDFLDDDNSRPYTYQKEKKSKNPN-KHISFKQRTVAYMEPFTLDVFISKRFVSASITHRVTSKQVAVAGTNS : 111
Heltu     : --MFKRACSM---------HGDDTAKLCLK-AIARHIHSAQARFGPRSFFGVEDFLDDDNSRPYTYQKEKKSKNPN-KHISFKQRTVAYMEPFTLDVFISKRFVSASITHRVTSKQVAVAGTNS : 111
Horvu     : --MLKNVLSQSWRRG--AHVLQEGHPAEALYACSSRFHSGQALRSSRSFFGVEDFMDEDNSKPYTYKKEKRSKNPH-KHISFKQRTIAHMEPFTLDVFISKRFVSASLTHRSTCRQVAVAGTNS : 119
Jughix    : --MLKYVLGKAWNKG-INGFGGDK-VRPPFFVLTSSFHNGQVHFAPRSFLGVEDFLDDDNSRPYTYQKGKKSKNPT-KHISFKQRTVAYMEPFTLDVFISKRFVSASLTHRVTSKQVAVAGTNS : 119
Lotja     : --MLKQAFGRICRTS-AYTCEG-NRVLPWIYVPSCFFHNGQVHMAPRSFFGVEDFLDDDNSRPYTYQKGKKSKNPN-KHVSFKQRTVAFMEPFTLDVFISKRFVSASVTHRVTSKQVAVAGTNS : 119
Manes     : --MLKQVLGKVLNKG-SCASGGDK-LRALFYVPITGFHNGQVYCAPRSFFGVEDFLDDDNSRPYTYQKEKKSKNPN-KHMSFKQRTKAYMEPFTLDILISKRFVTASLTHRVTSKQVAVAGTNS : 119
Medsa     : --MLKQAIGRICRTN-VYACEG-NNVLPWSYVPSCCFHNGQAHMAPRSFFGVEDFVDDDNSRPYTYQKGKKSKNPN-KHISFKQRTVAYMEPFTLDVFISKRFVSASLTHRVTSKQVAVAGTNS : 119
Medtr     : --MLKQAIGRICRTN-AYACEG-NKVLPWSYVSSCCFHNGQAHMAPRSFFGVEDFVDDDNSRPYTYQKGKKSKNPN-KHISFKQRTVAYMEPFTLDVFISKRFVSASLTHRVTSKQVAVAGTNS : 119
Mimgu     : --MLRLVCR-------KLGAHMNSGCNIRPLSQISGFHSSQTCLAPRSFFGVEDFLDDDNSRPYTYQKGKKSKNPN-KHVSFKQRTVAYMEPFTLDVFISKRFVSASITHRVTCKQVAVAGTNS : 114
Nicta     : --MLKLVSE-------ALCVRGGQTIRSVLCAQISGFHTAKPCLAPRSFFGVEDFLDDDNSRPYTYQKGKKSKNPN-KHVSFKQRTVAYMEPFTLDVFISKRFVSASITHRVTCKQVAVAGTNS : 114
Nupad     : --MLRNAFRNAWNGG---RLTSSEGGISSLFLATRGFHDGQVLLSPRSFFGVEDFVDDDNGRPYTYKKEKKSKNPH-KHASFKQRTIAYMEPFTLDVFISKRFVSASLTHRVTSKQVAVAGTNS : 118
Orysa     : --MLRNILCQSWRRG--AYALQEGNHPGALHACWSRFHSGQMLSSSRSFFGVEDFMDEDNSKPYTYKKEKRSKNPH-KHISFKQRTIAYMEPFTLDVFISKRFVSASLTHRTTCRQVAVAGTNS : 119
Phavu     : --MLKQAIGRICRTS-AYAFEG-NRVLPCIHVPSCFFHNGQTHMAPRSFFGVEDFLDDDNSRPYTYQKGKKSKNPH-KHISFKQRTIAYMEPFTLDVFISKRFVSASLTHRMTSKQVAVAGTNS : 119
Picgl     : --MSKLLLRDIWNKG---EHTLSRSHLDVFSVLAREIHSGKELCGPRSFFGVEDFVDDDNSKPYTYMKEKKSKNPH-KHVSFKQRTIAYMQPFTLDVFISKRFVSASITHRVTSKQVAVAGTNS : 118
Picsi     : --MFKLLLRDIWNKG---EHTLSRSHLDVFSVLAREIHSGKELCGPRSFFGVEDFVDDDNSKPYTYMKEKKSKNPH-KHVSFKQRTIAYMQPFTLDVFISKRFVSASITHRVTSKQVAVAGTNS : 118
Pinco     : --MFKRLLRDIWNKG---ENKLSRNHLDVFSVCAREFHSGKELCGPRSFFGVEDFVDDDNTKPYTYRKEKISKNPH-KHVSFKQRTIAYMQPFTLDVFISKRFVSASITHRVTSKQVAVAGTNS : 118
Pinpi     : --MFKRLLRDIWNKG---ENKLSRNHLDVFSVLAREFHSGKELCGPRSFFGVEDFVDDDNTKPYTYRKEKISKNPH-KHVSFKQRTIAYMQPFTLDVFISKRFVSASITHRVTSKQVAVAGTNS : 118
Pinta     : --MFKRLLRDIWNKG---ENKLRRNHLDVFSALAREFHSGKALCGPRSFFGVEDFVDDDNTKPYTYRKEKISKNPH-KHVSFKQRTIAYMQPFTLDVFISKRFVSASITHRVTSKQVAVAGTNS : 118
Popeu     : --MLKQVFGKVLNRR-FFVNGGDK-LRPGLYVPITGFHNGQAHCAPRSFFGVEDFLDDDNSRPYTYQKGKKSKNPN-KHLSFKQRTVAYMEPFTLDVFISKRFVSASLTHRVTSKQVAVAGTNS : 119
Poptr     : --MLKQVFGKVLNKG-FFANGGDK-LRPGLYVPNTGFHNGQAHCAPRSFFGVEDFLDDDNSRPYTYQKGKKSKNPN-KHLSFKQRTVAYMEPFTLDVFISKRFVSASLTHRVTSKQVAVAGTNS : 119
Quero     : --MLKHVLGKVCNRG-VNAYGGDK-FRPYLYVLTSDVHNGQVHCAPRSFFGVEDFLDDDNSRPYTYQKEKKSKNPN-KHVSFKQRTVAYMEPFTLDVFISKRFVSASLTHRVTSKQVAVAGTNS : 119
Rapsa     : --MWKQVVGKYSISK-AVYGNNRAKLQPFLCQTSCGFHNGRTVLAPRSFFGVEDYVDDDTSRPYTYQKEKKSKNPD-KHVSFKQRTIAYMEPFTLDVFISKRFVSASLTHRVTCKQVAVAGTNS : 120
Ricco     : --MLKQVLGKIIHRG-PFLNGGDK-LRLSLYVPGSCFHNGQVHYSPRSFFGVEDFLDDDNSRPYTYQKEKKSKNPNNKHMSFKQRTKAYMEPFTLDVFISKRFVSASLTHRVTSKQVAVAGTNS : 120
```

```
Saume      : --MLKRACSMS------YTHGSEKTKLCLK-AITRHFHSGQPHSAPRSFFGVEDFLDDDNSRPYTYQKEKKSKNPN-KHISFKQRTIAYMEPFTLDVFISKRFVSASLTHRVTSKQVAVAGTNS : 114
Setit      : --MLRNILSQSWRNG--AHVLQEGNPAPALYTCWRQFHSGQMLSSSRSFFGVEDFVDEDNSRPYTYKKEKRSKNPH-KHISFKQRTIAYMEPFTLDVFISKRFVSASLTHRSTCRQVAVAGTNS : 119
Solly      : --MLKLVSE-------MLCTRGGQKLRPYLCAQVSGFHTSKPALAPRSFFGVEDFVDDDNSRPYTYQKGKKSKNPN-KHVSFKQRTVAYMEPFTLDVFISKRFISASITHRVTCKQVAVAGTNS : 114
Soltu      : --MLKLVSE-------MLCVRGGQKMRPFLCAQISGFHTSKPGLAPRSFFGVEDFVDDDNSRPYTYQKGKKSKNPN-KHVSFKQRTVAYMEPFTLDVFISKRFVSASITHRVTCKQVAVAGTNS : 114
Sorbi      : --MLRSILSQSWKRG--AHMLCEGNSAPALLTCWSQFHSGQVLSSSRSFFGVEDFVDEDNSRPYTYKKEKRSKNPH-KHISFKQRTIAYMEPFTLDVFISKRFVSASLTHRSTCRQVAVAGTNS : 119
Strhe      : --MLRLSYR-------KLCARSNGGGEMMSLSQISSFHSSQTSLSPRSFFGVEDFLDDDNSRPYTYQKEKKSKTPN-KHVSFKQRTVAYMEPFTLDVFISKRFVSASITHRVTCKQVAVAGTNS : 114
Vitvi      : --MLKHLSGKLWIKG-VWANGRDQ-FRPCLYVPTSGFHNGKAHLAPRSFFGVEDFLDDDNSRPYTYQKEKKSKNPN-KHISFKQRTGAYMEPFTLDVLISKRFVSASLTHRVTSKQVAVAGTNS : 119
Zeama      : --MLKCILSQSWKRG--AHMLHAGNSAPAVLTCWSQFHSGQVWSSSRSFFGVEDFVDEDNSRPYTYKKEKRSKNPH-KHISFKQRTIAHMEPFTLDVFISKRFVSASLTHRSTCRQVAVAGTNS : 119
               m                                  fh         pRSFfGVED56DdDn 4PYTY K K 3Knp  KH SFKQRT A M2PF3LD6fIS4rF63As6THR T 4QVAVaGTNS

                   *       140           *       160           *       180           *
Aegta      : KDVKAALMSRSDVPACLSVGRFLAERAKEADVYACTYPPRERDRFEGKIRAVVQSLIDNGIHVKLYLD : 187
Araly      : KDVKAVLRSRCDIPACMSIGRILSERAKEADVYTASYTPRDQEKFEGKIRAVVQSLIDNGIDVKIYLD : 189
Arath_HES  : KDVKAVLRSRCDIPACMSIGRILSERAKEADVYTASYTPRDQDKFEGKIRAVVQSLIDNGIDVKIYLD : 189
Arahy      : KDIKAVFKSRSDIPACIAIGRILAERAREADVYTASYTPRDRDKFEGKIRAVVQSLIDNGIDVKVYLD : 191
Aspof      : KDVKAALQSRSDIPACLAVGRILAERAREADVYTATYTPRERDRFEGKIRAVVQSLIDNGIDVKVYLN : 182
Bradi      : KDVKAALASRSDVPACLSVGRFLAERAKEADVYACTYTPRERDRFEGKIRAVVQSLIDNGIHVKVYLD : 187
Brana      : KDVKAVLRSRCDIPACMSIGRILSERAKEADVYTASYTPRDQDKFEGKIRAVVQSLIDNGIDVKIYLD : 185
Braol      : KDVKAVLRSRCDIPACMSIGRILSERAKEADVYTASYTPRDQDKFEGEIRAVVQSLIDNGIDVKIYLD : 185
Cenma      : KDIKAALKSRSDIPACLAIGKILSERAIEADVFTASYTPRERDKFEGKIRAVVQSLIDNGIDVKIYLD : 182
Cicar      : KDIKAVLRSRSDIPACIAIGRILAERAREADVYTASYTPRDRDKFEGKIRAVVQSLIDNGIDLKVYLD : 187
Cicen      : KDIKAALQSRSDIPACLAIGRILSERAREADVYTASYTPRERDKFEGKIRAVVQSLIDNGIDVKIYLD : 182
Citcl      : KDIKAVLRSRSDIPACLAIGRILAERAREADVYTASYTPRERDKFEGKIRAVVQSLIDNGIDVKIYLD : 188
Cofca      : KDIKAVLKSRSDIPACLAIGRILAERAREADVYTASYTPRDRDKFEGKIRAVVQSLIDNGIDVKVYLE : 164
Cycru      : KHIKAVLRSKSDIPACLAVGQILAERSREADVFTASYTPRERDKFEGKIRAVVQSLIDHGIDIKVYLN : 186
Elagu      : KDIKAALQSRSDIPACLAIGRILAERAREADVYTCTYTPRDSDKFEGKIRAVVQSLIDNGIDVKVYLD : 186
Eutsa      : KDVKAVLRSRCDIPACMSIGRILSERAKEADVYTASYTPRDQDKFEGKIRAVVQSLIDNGIDVKIYLD : 188
Glyma      : KDIKAVLRSRSDIPACIAIGRILAERAKEADVYTGSYTPRDRDKFEGKIRAVVQSLIDNGIDIKVYLD : 187
Gosra      : KDIKAVLRSRSDIPACLAIGRILAERAREADVYTASYTPRERDKFEGKIRAVVQSLIDNGIDIKVYLD : 188
Helan      : KDIKAALKSRSDIPACLAXGRILSDRAREADVYTASYTPRERDKFEGKIRAVVQSLIDNGIDVKIYLD : 183
Helci      : KDIKAELKSRSDIPACLAIGRILSQRAREADVYTASYTPRERDKFEGKIRAVVQSLIDNGIDVKIYLD : 179
Heltu      : KDIKAALKSRSDIPACLAIGRILSQRAREADVYTASYTPRERDKFEGKIRAVVQSLIDNGIDVKIYLD : 179
Horvu      : KDVKAALTSRSDVPACLSVGRFLAERAKEADVYACTYTPRERDRFEGKIRAVVQSLIDNGIHVKLYLD : 187
Jughix     : KDIKAVLKSRSDIPACLAVGAILADRAREADVYTASYTPRERDKFEGKIRAVVQSLIDNGIDVKIYLD : 187
Lotja      : KDIKAVLRSRSDIPACIAIGRILAERAREADVYTASYTPRDRDKFEGKIRAVVQSLIDNGIDIKVYLD : 187
Manes      : KDIKAALKSRSDIPACLAIGRILADRAREADVYTASYTPRERDKFEGKIRAVVQSLIDNGIDVKVYL- : 186
Medsa      : KDIKAVLRSRSDIPACIAIGRILAERAMEADVYTASYTPRERDKFEGKIRAVVQSLIDNGIDLKVYLD : 187
Medtr      : KDIKAVLRSRSDIPACIAIGRILAERAMEADVYTASYTPRDRDKFEGKIRAVVQSLIDNGIDLKGYLD : 187
Mimgu      : KDIKAALRSRSDIPACLSVGRILSDRAREADVYTASYTPRERDKFEGKIRAVVQSLIDNGIDIKIYLD : 182
Nicta      : KDIKAVLKSRSDIPACLSVGQILADRAREADVYTASYTPRDRDKFEGKIRAVVQSLIDNGIDIKVYLD : 182
Nupad      : KGIKAILRSRCDIPACLAVGQILAERAREADVYIASYTPRDRDKFEGKIRAVVQSLIDNGIDVKVYLN : 186
Orysa      : KDIKAALKSRSDIPACLAVGRFLAERAKEADVYTCTYTPRERDKFEGKIRAVVQSLIDNGINVKVYLD : 187
Phavu      : KDIKAVLRSRSDIPACISVGKILSERAREADVYTASYTPRERDKFEGKIRAVVQSLIDNGIDVKVYLD : 187
```

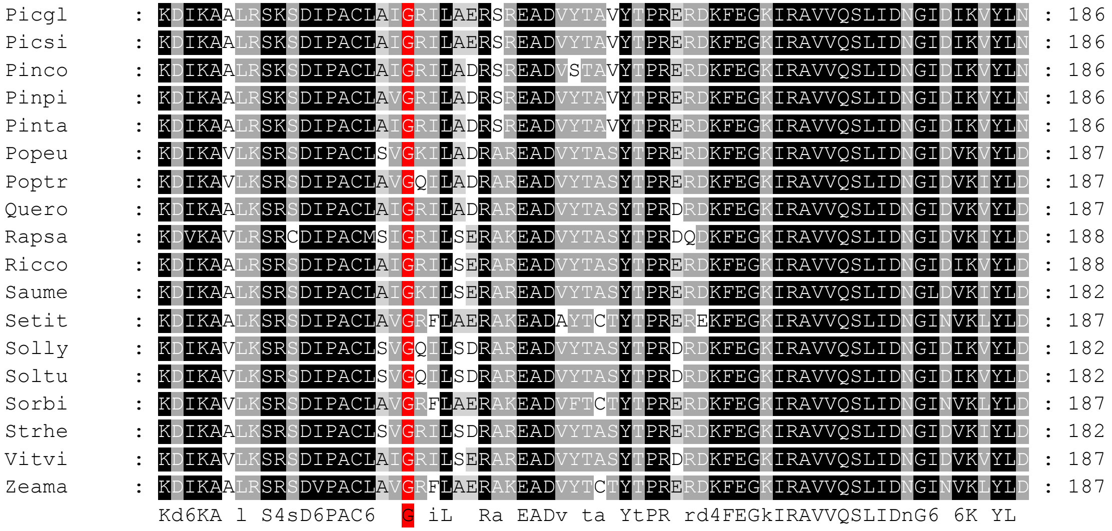

Supplemental Figure S3. Clustal alignment of amino acid sequences of putative plant HES orthologs.

Consensus sequence similarity is shown by shading from black, most similar (identical) through grey to white showing no consensus similarity. The glycine-141 mutated in the hes protein is highlighted in red in all sequences. The sequences are identified by a five letter acronym comprising the first three letters of the genus name followed by the first two letters of the species name. The species and sequence IDs used for this figure are listed below in alphabetical order: *Aegilops tauschii* (EMT21903), *Arabidopsis lyrata* (XP_002889717.1), *Arabidopsis thaliana* (At1g08845), *Arachis hypogaea* (GO340846), *Asparagus officinalis* (CV289985), *Brachypodium distachyon* (XP_003574328), *Brassica napa* (ES922646), *Brassica oleracea* (DY028966), *Centaurea maculosa* (EH743846), *Cicer arietinum* (XP_004499792.1), *Cichorium endivia* (EL365047), *Citrus clementina* (XP_006433150.1*), Coffea canephora* (DV694863), *Cycas rumphii* (EX924437), *Cynara cardunculus* (GE592927), *Elaeis guineensis* (EY405582), *Eutrema salsugineum* (XP_006398012.1*), Glycine max* (XP_003522852.1), *Gossypium raimondii* (CO071140*), Helianthus annuus* (GE516931), *Helianthus ciliaris* (EL431545), *Helianthus tuberosus* (EL461258), *Hordeum vulgare* (BAK01660.1), *Juglans hindsii x Juglans regia* (EL900814), *Lotus japonica* (FS328398), *Manihot esculenta* (DV455681), *Medicago sativa* (EX523670), *Medicago truncatula* (BF648661*), Mimulus guttatus* (GO969360), *Nicotiana tabacum* (FS433097), *Nuphar advena* (ES734632), *Oryza sativa* (NP_001065375), *Phaseolus vulgaris* (AGV54630.1, *Picea glauca* (EX395862), *Picea sitchensis* (ABK25907.1*), Pinus contorta* (GT248182), *Pinus pinaster* (AL750631*), Pinus taeda* (DR052843), *Populus euphratica* (AJ769277), *Populus trichocarpa* (XP_002319579), *Quercus robur* (FN749455), *Raphanus sativus* (FD950087), *Ricinus communis* (XP_002534606), *Saussurea medusa* (FC860339_1), *Setaria italica* (XP_004951444.1), *Solanum lycopersicum*

(XP_004237376.1*), Solanum tuberosum* (XP_006364507.1), *Sorghum bicolour* (XP_002467495), *Striga hermonthica* (FS491702), *Vitis vinifera* (XP_002266607.2), *Zea mays* (NP_001143121). Most sequence IDs refer to an EST sequence that was translated in the correct reading frame to provide the HES ortholog protein sequence.

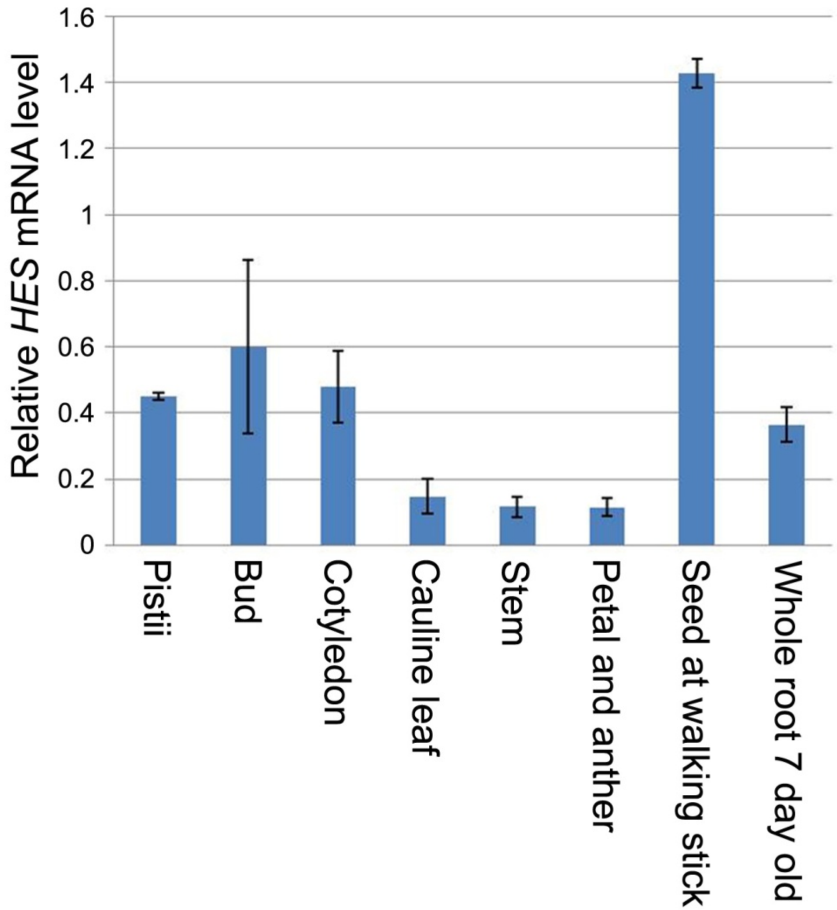

Fig. S4. Q-PCR with the *HES* gene in various plant tissues
