# Supplementary material for: Developmentally regulated HEART STOPPER, a mitochondrially targeted L18 ribosomal protein gene, is required for cell division, differentiation, and seed development in Arabidopsis
Source: J Exp Bot. 2015 Jun 23;66(19):5867–80. doi: 10.1093/jxb/erv296 (PMC4566979; doi:10.1093/jxb/erv296)
Supplement: Supplementary Data [file supp_erv296_JEXPBOT_145201_Supplementary_Methods.pdf]

Supplementary methods

Materials, growth conditions, genetic mapping, gene cloning, phylogenetic analysis, reporter gene constructs, gene expression

Materials and growth conditions

L*er*, Col, *MEA:GUS*, *FIS2:GUS*, and *MINI3:GUS* have been used in this experiments (Luo et al., 2000 and 2005). The MEA:GUS, FIS2:GUS, and MINI3:GUS reporter lines were crossed to *HES/hes* for progeny with markers expressed in the *hes* heterozygotes.

T-DNA insertion lines for the linked genes of *HES* were obtained from the *Arabidopsis* Stock centre (http//:www.arabidopsis.org).

All plants were grown in pots with compost soil under continuous artificial light at 20°C in LB growth chambers. The artificial light was achieved by using an incandescent light source producing 150 μmol photons.m^{-2}.sec^{-1}

Genetic mapping, mutation detection and complementation

HES/hes was crossed to Columbia. In the F1 population, only the heterozygotes were harvested for further analysis. As homozygous *hes/hes* plants did not occur, the genotype of each F2 plant was scored with seed phenotypes. Selected SSLP markers (http//:www.arabidopsis.org) from five chromosomes were used to determine the genotypes for each F2 plant and the map location of *HES* was able to be calculated based on the association between seed phenotypes and markers. The *HES* gene was located close to a SSLP marker F16J7-TRB (http//:www.arabidopsis.org) on Chromosome 1. For detailed mapping and cloning, a range of indel markers covering the *HES* region were designed based on the Monsato SNP data base (http//:www.arabidopsis.org) (Table S11). The genetic distance as shown by the crossover frequency (total crossovers out of total chromosomes) for each marker is indicated in Fig. S2. Primers for mapping are listed in Table S11. The *HES* is located between 2764k FR and 2867k FR, a 103kb region containing 25 annotated genes (At1g08700 to At1g08960; www.arabidopsis.org). Lines with T-DNA insertion in exons of 16 of the 25 genes were examined but no *hes*-like seed phenotypes were observed.

We then PCR amplified the other nine candidate genes from heterozygous *HES/hes* plants into individual overlapping fragments. A heteroduplex DNA PCR fragment between WT and *hes* DNA should have a mismatch that can be cleaved by the nuclease CEL1 at the mismatch site (Oleykowski

et al., 1998). The PCR fragments, after denaturing and annealing, were digested by adding 0.5µl CEL1 enzyme mixture for 30 minute at 40ºC and visualized on a 1.5% Agarose gel. The PCR fragments overlapped each other by at least 200 bp to avoid the mismatch site being too close to the end of a particular fragment. A mismatch was identified from a fragment derived from At1g08845 and confirmed by Sanger sequencing (Fig. S2B). The At1g08845 gene contains two introns and produces a ~700 nucleotide transcript based on ESTs from this region of the genome. The longest transcript contains a 179 nucleotide 5' UTR, a 39 nucleotide 3'UTR with a 567 nucleotide coding region. Although another At1g08845 transcript was predicted (longer at the 5' end with two additional introns and an extra 40 amino acids), this was not supported by ESTs or by comparison to the deduced sequences of orthologs in other plant species (Fig. S3). The *hes* mutant contains a single nucleotide substitution of G to A at nucleotide 421 of the At1g08845 coding region, which is predicted to lead to a conserved glycine (G) at position 141 being replaced by an arginine (R) (Fig. S3).

For complementation, a DNA fragment containing only the At1g08845 L*er* genomic sequence from 798 nucleotides upstream of the longest *HES* EST and 283 nucleotides downstream of the stop codon was amplified using the primers: CTTGGGCTTCTTTCTTGGTG and GAGGTGGCATTTTCTCTGAGTT, cloned in pGEM T-Easy plasmid (Promega) and finally the *Not*1 fragment transferred to the binary plasmid pART27. The upstream primer used for cloning HES promoter locates in the coding region of the upstream gene At1g08840, thus this promoter should be a full length. This complementation construct and the *HES:GFP* construct below include the coding region of a longer annotation of the At1g08845 gene that is not supported by EST evidence nor by comparison of the deduced sequences of orthologs in other plant species.

The plants derived from *HES/hes* heterozygous plants were floral dipped with Agrobacterium stain GV3101 containing binary plasmids carrying At1g08845 genomic sequence (Clough and Bent, 1998). Primary transformants were selected on MS medium supplemented with 50µg/ml Kanamycin. Three types of transgenic kanamycin-resistant T1 plants were obtained (Table S1). Type I plants, like wild type, had no small-sized aborted seeds; type II showed reduced ratios of small seeds compared to *HES/hes* (Fig. S2C); and type III showed the ratio of small size seeds comparable to *HES/hes* (Fig. S2C). Sequencing of type II and III plants showed both had the A to G mutation (Fig. S2C). We reasoned that the type III plants were homozygous *hes* mutants and hemizygous for the transgene, and type II plants were either *HES/hes* heterozygotes and hemizygous for the transgene, or homozygous *hes/hes* with multi-copies of transgenes. We then grew seeds from one T1 plant of the type III for progeny analysis. If the type III is homozygous for the *hes* mutation and hemizygous for the complementing transgene, we expected that all the T2 plants should survive kanamycin selection because homozygous T1 seeds without the complementing transgene were aborted and not able to germinate. All 600 plants were resistant to kanamycin. We then analyzed the progeny of a type II T1 plant. By growing the seeds on selection, we obtained plants showing phenotypes of type I (51

plants), type II (33 plants) and type III (14 plants), close to the expected 3:2:1 ratio (Chi-square=0.409; p 0.815). All T3 seedlings from two type III plants in this population were also kanamycin resistant. These results indicated that At1g08845 complements the *hes* mutation and encodes the *HES* gene.

Phylogenetic analysis

The amino acid sequences of ribosomal L18/L5 family proteins were obtained by blasting from the NCBI database (http://www.ncbi.nhn.nih.gov) using the HES protein sequence. Mega4.1 was used to make sequence alignments and construct phylogenetic trees using the Neighbour Joining algorithm. The protein IDs (or accession numbers) and species names can be identified in the phylogenetic tree.

Reporter gene constructs: pHES:HES:GFP and pHES:HES:GUS

The pGEM T-Easy plasmid carrying the complementing At1g08845 genomic sequence was used as a PCR template using primers: CGCGGATCCTAGAAAACAAATGTTTGTTGTGTCCGTG

and CCGCTCGAGATCGAGATAAATTTTAACATCAATTCCGTT, that amplify the whole plasmid and insert *Xho* I and *Bam* HI sites immediately upstream of the *HES* stop codon at the ends of the essentially linearised complementation plasmid PCR product. An *Xho* I /*Bam* HI *GFP* fragment removed from a *GFP* pBIN19 derivative was ligated to the *Xho* I / *Bam* HI digested amplified complementation plasmid resulting in an in-frame fusion of GFP to the C-terminus of the complete HES polypeptide. The resulting recombinant plasmids were selected on Amp LB plates and then sequenced for the insertion of *GFP* in front of the stop codon of At1g08845 (pGEM HES:GFP). The verified plasmids carrying *HES:GFP* fusion was cut with *Not* I and the insert was transferred to pART 27, a binary plasmid for plant transformation. The *HES/hes* heterozygous plants were floral dipped with Agrobacterium stain GV3101 containing binary plasmids carrying HES:GFP genomic sequence. Three types of transgenic kanamycin-resistant T1 plants were also obtained (Table S1). Type I plants, like wild type, had no small-sized aborted seeds; type II showed reduced ratios of small seeds compared to *HES/hes* (Fig. 5A); and type III showed the ratio of small size seeds comparable to *HES/hes*. The plants showed reduced formation of aborted seeds were used for analysis.

Similarly, we amplified *GUS* fragments from plasmid DNA pBI101-2 using primers ACCGCTCGAGATGTTACGTCCTGTAGAAACCCCAA and CGCGGATCCTCATTGTTTGCCTCCCTGCTG. These amplified GUS fragments and pGEM *HES:GFP* were cut with *Xho* I and *Bam* HI and ligated to obtain pGEM *HES:GUS*. The verified plasmids carrying *HES:GUS* fusion was cut with *Not* I and the insert was transferred to pART 27. The L*er* was floral dipped with Agrobacterium stain GV3101 containing binary plasmids carrying

HES:GUS genomic sequence.

Quantitative RT-PCR

Wild type L*er* RNAs were extracted from different tissues at least three times, using Qiagen RNeasy Plant Mini kits. cDNAs were synthesized using SuperScript III. qRT-PCR was conducted on cDNAs derived from three independent RNA samples. *HES* coding region-specific primers are CAATGGACGGACTGTTTTTGCA and TGCTTGAACGACACATGCTTG. Reference gene *ACTIN 2* (At3g18780) primers are CGCTCTTTCTTTCCAAGCTCAT and TCCTGCAAATCCAGCCTTC. The homozygous plants carrying *HES:GUS* were used for examining HES expression in more details.

Twenty randomly-selected genes with decreased expression and eighteen up-regulated genes were used for verification of microarray results. Reference genes *ACTIN 2* , *APC2* (At2g04660) and *HBT* (At2g20000) showed stable expression in *hes* and wt seeds in microarray assay (Table S2). Seven up-regulated genes in "mitochondrial dysfunction regulon" were verified with the three reference genes (Table S5). The rest were only tested with *ACTIN2*.

References

Clough SJ, Bent AF. 1998, Floral dip: a simplified method for Agrobacterium-mediated transformation of *Arabidopsis thaliana*. *The Plant Journal* **16**, 735–743.

Luo M, Bilodeau P, Dennis ES, Peacock WJ, Chaudhury A. 2000, Expression and parent-of-origin effects for FIS2, MEA, and FIE in the endosperm and embryo of developing Arabidopsis seeds. *Proceedings of the National Academy of Sciences* **97**, 10637–10642.

Luo M, Dennis ES, Berger F, Peacock WJ, Chaudhury A. 2005, MINISEED3 (MINI3), a WRKY family gene, and HAIKU2 (IKU2), a leucine-rich repeat (LRR) KINASE gene, are regulators of seed size in Arabidopsis. *Proceedings of the National Academy of Sciences of the United States of America* **102**, 17531–17536.

Oleykowski CA, Mullins CRB, Godwin AK, Yeung AT. 1998, Mutation detection using a novel plant endonuclease. *Nucleic Acids Research* **26**, 4597–4602.
